# A systematic evaluation of source reconstruction of resting MEG of the human brain with a new high‐resolution atlas: Performance, precision, and parcellation

**DOI:** 10.1002/hbm.25578

**Published:** 2021-07-05

**Authors:** Luke Tait, Ayşegül Özkan, Maciej J. Szul, Jiaxiang Zhang

**Affiliations:** ^1^ Cardiff University Brain Research Imaging Centre Cardiff University Cardiff

**Keywords:** cortical atlas parcellation, Magnetoencephalography, resolution analysis, resting‐state, source reconstruction

## Abstract

Noninvasive functional neuroimaging of the human brain can give crucial insight into the mechanisms that underpin healthy cognition and neurological disorders. Magnetoencephalography (MEG) measures extracranial magnetic fields originating from neuronal activity with high temporal resolution, but requires source reconstruction to make neuroanatomical inferences from these signals. Many source reconstruction algorithms are available, and have been widely evaluated in the context of localizing task‐evoked activities. However, no consensus yet exists on the optimum algorithm for resting‐state data. Here, we evaluated the performance of six commonly‐used source reconstruction algorithms based on minimum‐norm and beamforming estimates. Using human resting‐state MEG, we compared the algorithms using quantitative metrics, including resolution properties of inverse solutions and explained variance in sensor‐level data. Next, we proposed a data‐driven approach to reduce the atlas from the Human Connectome Project's multi‐modal parcellation of the human cortex based on metrics such as MEG signal‐to‐noise‐ratio and resting‐state functional connectivity gradients. This procedure produced a reduced cortical atlas with 230 regions, optimized to match the spatial resolution and the rank of MEG data from the current generation of MEG scanners. Our results show that there is no “one size fits all” algorithm, and make recommendations on the appropriate algorithms depending on the data and aimed analyses. Our comprehensive comparisons and recommendations can serve as a guide for choosing appropriate methodologies in future studies of resting‐state MEG.

## Graphical abstract

Magnetoencephalography (MEG) measures extracranial magnetic fields, requiring source reconstruction to make neuroanatomical inferences from these signals. Here, we evaluated the performance of six commonly‐used source reconstruction algorithms based on minimum‐norm and beamforming estimates in the context of human resting‐state MEG. Our results show that, there is no “one size fits all” algorithm, and make recommendations on the appropriate algorithms depending on the data and aimed analyses.

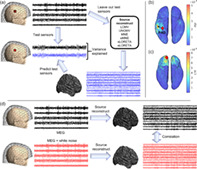

## 1 INTRODUCTION

Noninvasive imaging of the human brain's resting‐state dynamics can give insight into the neuronal mechanisms underpinning healthy cognition and neurological disorders (Babiloni et al., 2016; Cohen, 2018; Douw et al., 2011; Lee, Moser, Ing, Doucet, & Frangou, 2019; Michel & Koenig, 2018; van den Heuvel & Hulshoff Pol, 2010). Hemodynamic or metabolic correlates of resting‐state neuronal activity such as functional MRI (fMRI; Greicius, Krasnow, Reiss, & Menon, 2003) or positron emission tomography (Raichle et al., 2001) have identified key brain networks exhibiting spontaneous functional connectivity during rest, but their temporal resolutions are much slower than the timescale of neuronal oscillations. Hence, properties such as oscillatory power spectra and phase‐binding (Babiloni et al., 2016), and the brain's rapid transitions in active networks at the temporal order of tens to hundreds of milliseconds (Baker et al., 2014; Michel & Koenig, 2018) cannot be studied using these modalities. Conversely, conventional magneto‐ or electro‐encephalography (M/EEG) measure macroscopic neuronal postsynaptic activity at a high temporal resolution of the order of milliseconds, but sensors are located outside of the head and cannot directly infer the spatial origin of signals.

To make neuroanatomical inferences on the M/EEG signal, source reconstruction must be used (Michel et al., 2004; Michel & Brunet, 2019). This problem is ill‐posed and therefore has no unique solution, but the neurophysiologically‐informed assumptions underpinning M/EEG signals can be used to constrain the solution (by fixing dipole locations, orientations, etc.). As a result, a wide range of algorithms exist to solve this inverse problem based on different assumptions and priors (Dale et al., 2000; Friston et al., 2008; Fuchs, Wagner, Köhler, & Wischmann, 1999; Hämäläinen & Ilmoniemi, 1994; Huang et al., 1998; Mosher & Leahy, 1998; Pascual‐Marqui, 2002, 2007; Pascual‐Marqui, Michel, & Lehmann, 1994; Van Veen, van Drongelen, Yuchtman, & Suzuki, 1997). Much of the literature has assessed the ability of different inverse algorithms in identifying sources of task‐evoked activity (Bai, Towle, He, & Bin, 2007; Halder, Talwar, Jaiswal, & Banerjee, 2019; Hassan, Dufor, Merlet, Berrou, & Wendling, 2014; Seeland, Krell, Straube, & Kirchner, 2018) or localizing simulated dipoles (Anzolin et al., 2019; Barzegaran & Knyazeva, 2017; Bonaiuto et al., 2018; Bradley, Yao, Dewald, & Richter, 2016; Finger et al., 2016; Halder et al., 2019; Hassan et al., 2017; Hincapié et al., 2017; Pascual‐Marqui et al., 2018).

The current study aims to address three challenges in magnetoencephalography (MEG) source reconstruction. First, is there an optimal inverse algorithm to use for resting‐state MEG data? The answer is not trivial, because resting‐state activity may exhibit different spatiotemporal characteristics than task‐evoked activity in the source space. Furthermore, the signal‐to‐noise ratio (SNR) of resting‐state MEG data is often lower than that of evoked activity because of the inability to average over multiple trials to reduce noise (Parkkonen, 2010). Here, we provide a comprehensive assessment on six widely used source‐reconstruction algorithms in analyzing resting‐state data: the linearly constrained minimum variance (LCMV) beamformer (Van Veen et al., 1997) and its depth normalized counterpart (unit‐noise‐gain LCMV, Sekihara & Nagarajan, 2015), three methods based on least‐squares minimum norms under different prior assumptions of source covariance, namely the minimum norm estimate (MNE) (Hämäläinen & Ilmoniemi, 1994), weighted MNE (Fuchs et al., 1999; Lin et al., 2006), and eLORETA (Pascual‐Marqui, 2007, 2009)), as well as a variance‐normalized MNE, sLORETA (Pascual‐Marqui, 2002).

For each algorithm, we evaluated both spatial and temporal metrics to assess its performance and precision to reconstruct distributed single‐trial MEG using resting‐state data. Spatial metrics involve the theoretical resolution properties (Hauk, Stenroos, & Treder, 2019; Hauk, Wakeman, & Henson, 2011; Hedrich, Pellegrino, Kobayashi, Lina, & Grova, 2017). The temporal metric refers to the cross‐validated temporal variance of sensor space data explained by the source estimates, quantifying whether the temporal fluctuations in source estimates accurately recreate those in the underlying brain dynamics. The temporal metric in the current study is related to that of Little et al. (2018). However, our metric is expressed as a gain versus empty room data, such that overfitting to data with non‐neuronal origin is penalized.

Second, do different source‐reconstruction algorithms lead to similar solutions, and how are they affected by external noise? Little work has addressed the similarity (or dissimilarity) of the spatiotemporal patterns of resting‐state source estimates from different algorithms. The answer to this question is of particular importance, as it allows for comparison between studies using different algorithms, and may act as a point of reference in terms of reproducibility when choosing a source algorithm for a study. Additionally, in typical task‐based analysis, the sensor‐level MEG data can be averaged over many trials to reduce the sensor‐level noise, and hence only minimal sensor noise is projected to sensor space. For resting‐state analysis, data averaging in the sensor space is not commonly performed; hence, sensor noise will have a greater influence over the source‐space solution. The projected sensor‐noise may bias the estimates of statistics and connectivity, even if the statistics are subsequently averaged over short epochs in source‐space. In the context of single‐trial distributed activity there is limited work addressing the extent to which source estimates are biased by external noise. These questions are addressed here via spatiotemporal correlation of source solutions, either between the six algorithms combined with spectral clustering for the former, or within each algorithm after the addition of artificial noise for the latter.

Third, we present a data‐driven approach to obtain a reduced variant of the Human Connectome Project Multimodal Parcelation (HCP‐MMP) atlas (Glasser et al., 2016), making it specifically suitable for MEG source reconstruction. Our updated atlas hence complements many existing human brain atlas that are derived from MRI or histological data (Desikan et al., 2006; Destrieux, Fischl, Dale, & Halgren, 2010; Fan et al., 2016; Tzourio‐Mazoyer et al., 2002).

In source reconstruction analyses, it is typical to parcellate the source‐localized data into a number of regions of interest (ROIs), for example, inability to average over many trials for measuring functional connectivity (Brookes et al., 2016; Colclough et al., 2016; Dauwan et al., 2019; Tewarie et al., 2014). Recently, there is a surge of interest in applying the HCP‐MMP atlas (Glasser et al., 2016) for parcellation of cortical dynamics (Dermitaş et al., 2019; Dubois, Galdi, Paul, & Adolphs, 2018; Ito et al., 2017; Preti & Van De Ville, 2019; Watanabe, Rees, & Masuda, 2019). The ROI boundaries in the HCP‐MMP atlas are defined by combined characteristics in cortical architecture, function, connectivity, and topography, offering better neuroanatomical precision and functional segregation. However, the HCP‐MMP atlas consists of 360 cortical ROIs, and the use of such a high‐resolution atlas is problematic for MEG due to its limited spatial resolution. For example, the current generation of MEG scanners has 200–300 sensors (McCubbin et al., 2004). Hence, parcellation of source space data into more than this number of ROIs results in rank deficiency. This is particularly an issue if one aims to measure functional networks, since multivariate orthogonalization, which is often used to reduce spurious correlations due to source leakage (Colclough, Brookes, Smith, & Woolrich, 2015), requires full rank data. Here, we present a reduction of the HCP‐MMP atlas with 230 ROIs, based upon the forward transformation between source dynamics and MEG sensors. Since parcellation greatly reduces the data dimension, and previous studies of single‐trial/resting‐state MEG have predominantly focused on comparing algorithms at the voxel‐level (Hauk et al., 2011; Hauk et al., 2019; Hedrich et al., 2017; Little et al., 2018; Liu, Ganzetti, Wenderoth, & Mantini, 2018; Seeland et al., 2018), we further performed resolution and variance‐explained analyses to examine the extent to which voxel‐level comparisons between the six algorithms remain consistent when estimated source data is parcellated using our high‐resolution atlas.

The results of this study could serve as a methodological guide for (a) choosing appropriate source reconstruction algorithms for analysis of resting‐state MEG, and (b) deriving appropriate atlases for parcellation of resting‐state MEG functional dynamics and connectivity. To facilitate these contributions, we have made our analysis scripts and the MEG‐optimized HCP‐MMP atlas open‐source and freely available (https://github.com/lukewtait/evaluate_inverse_methods).

## 2 MATERIALS AND METHODS

### 2.1 Source reconstruction algorithms

Many methods exist for source reconstructing M/EEG data. All methods discussed in the current study involve inverting a *forward model*,

(1) x=Ls+η,

where $x\in {\mathbb{R}}^{N_{x}\times T}$ is the empirical M/EEG data (here, $N_{x}$ is the number of gradiometers/electrodes, and T is the number of sampling points), $s\in {\mathbb{R}}^{N_{s}\times T}$ is the source data (here, $N_{s}$ is the number of dipoles), $\eta \in {\mathbb{R}}^{N_{s}\times T}$ is a noise term, and $L\in {\mathbb{R}}^{N_{x}\times N_{s}}$ is the scalar leadfield matrix (i.e., dipole orientations are fixed). Details of leadfield matrix construction for this study are outlined in Section 2.6, and a more general review of methods is given by Hallez et al. (2007). For resting‐state data, dipoles are generally distributed evenly across the grey matter of the cortical surface to construct the leadfield (Dale et al., 2000; Hillebrand & Barnes, 2003). Source data can then be estimated as

(2) $$\hat{s}=\Phi x,$$

where $\Phi \in {\mathbb{R}}^{N_{s}\times N_{x}}$ is an inverse matrix. Rows of Φ are often called spatial filters. Algorithms to estimate Φ can be separated into beamforming and least‐squares minimum norm (LSMN) type estimates. Below, we briefly introduce the algorithms considered in the current study. The mathematical details of each of these solutions are provided in Appendix A.

Beamforming involves scanning over each dipole in the source space individually, calculating the corresponding spatial filter independently of all other dipoles. The LCMV beamformer (Van Veen et al., 1997) requires that the solution has unit gain (i.e., $\Phi_{i}L_{i}=1$), and that cross talk from other locations is minimized (i.e., $\Phi_{i}L_{j}$ is minimized for all j≠i). This filter is known to mislocalize superficial power to deep sources. To account for this, the beamformer weights can be normalized (Van Veen et al., 1997) to unit vector norm, called a *unit‐noise‐gain* minimum variance beamformer (UNGMV; Sekihara & Nagarajan, 2015).

While beamformers consider each source point individually, regularized LSMN type solutions reconstruct all sources within the source space simultaneously, by minimizing the difference between the data x and the predicted data $L\hat{s}$, subject to regularization. A number of solutions have been proposed, differing in the prior estimate of source covariance, W. The simplest solution, often known as the MNE (Hämäläinen & Ilmoniemi, 1994), sets W=I. Much like the LCMV beamformer, the MNE solution suffers from depth bias, so the *weighted minimum norm estimate* (wMNE) sets the diagonals of W inversely proportional to the norm of the leadfield (Fuchs et al., 1999; Lin et al., 2006), essentially assuming a priori that sources which only weakly influence the M/EEG must have a higher variance to be measured by the sensors. The *exact low resolution electromagnetic tomography* (eLORETA) solution (Pascual‐Marqui, 2007, 2009) extends this assumption further, using an iterative algorithm to optimize the diagonals of W such that not only is the depth bias accounted for, the solution attains theoretically exact localization (Pascual‐Marqui, 2007).

Finally, *standardized low resolution electromagnetic tomography* (sLORETA; Pascual‐Marqui, 2002), an alternative approach to account for depth bias in the source localization, is to estimate *standardized* distributions of current density normalized by expected variance of each source. sLORETA first calculates the MNE solution, and then normalizes each time point to unit theoretical variance. This standardization reduces the depth bias and also has theoretically exact localization, but the resulting solution is no longer a measure of current density, and is more appropriately interpreted as the probability of source activation.

Note that all the six algorithms considered here make prior estimates of source variance, but make no prior estimates of source covariance, since the matrices W are always diagonal. For all source reconstruction algorithms, we used the implementations in Fieldtrip (Oostenveld, Fries, Maris, & Schoffelen, 2011; http://www.ru.nl/neuroimaging/fieldtrip, version July 16, 2019).

#### 2.1.1 Regularization

All the source reconstruction algorithms considered here can be regularized with a regularization parameter λ (Appendix A). For a fair comparison of algorithms, it is important to select λ appropriately and comparably across all algorithms. As highlighted in Appendix A.3, λ is related to the predicted SNR of the data (Hauk et al., 2019; Lin, Witzel, Zeffiro, & Belliveau, 2008). Figure S1e–g showed that 2.5 dB is an appropriate SNR level of our data. When presenting our main results, we therefore set the regularization parameter λ of each algorithm corresponding to SNR = 2.5 dB unless otherwise stated. Furthermore, to evaluate to what extent algorithm comparisons are influenced by under‐ or overestimation of SNR, we repeated all our main analyses with a wide range of predicted SNR values from −10 to 10 dB.

### 2.2 Comparing algorithms

**FIGURE 1:**
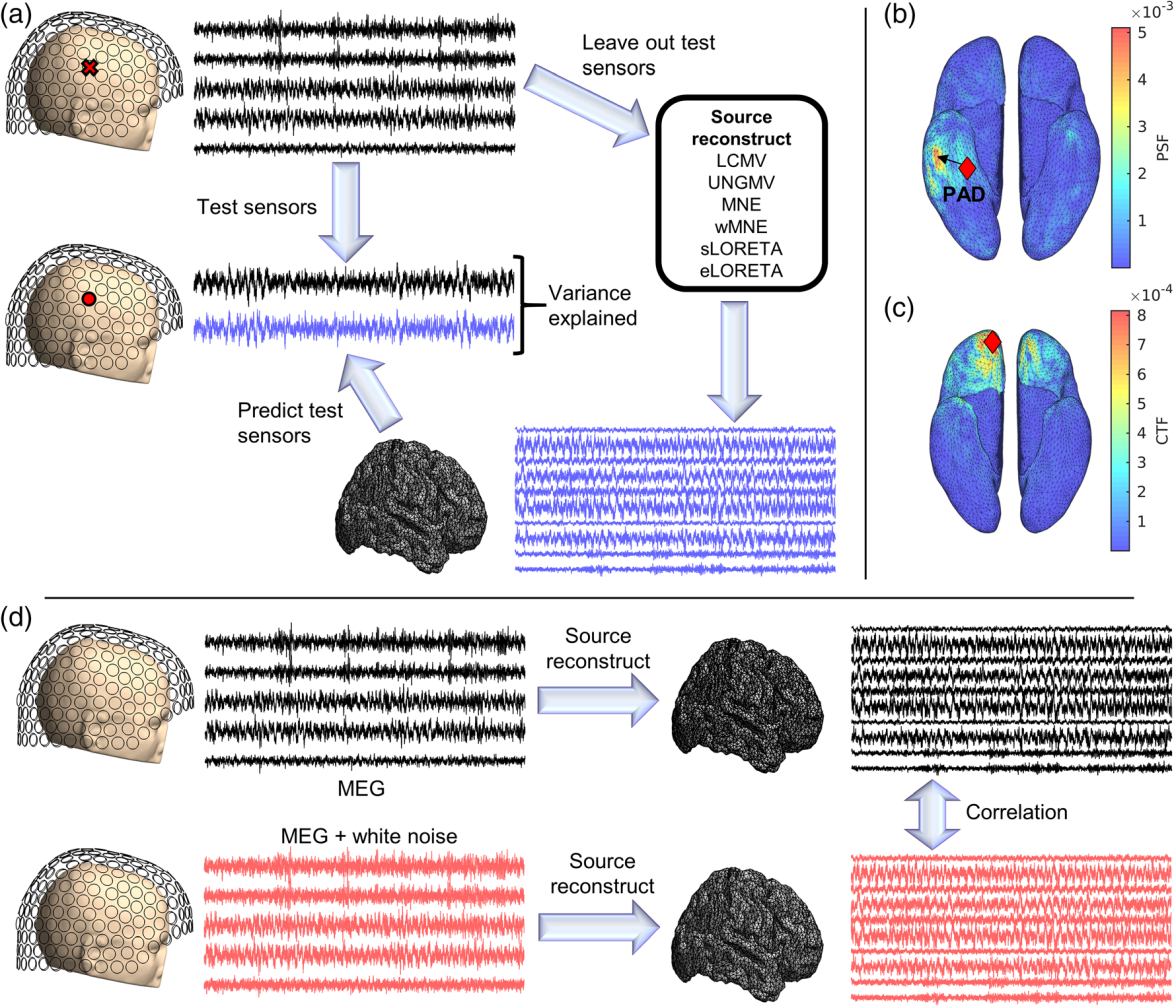
Variance explained analysis methods of comparing source reconstruction algorithms. (a) Variance explained analysis. Two hundred seventy‐four channel MEG data are split into 27 partitions of 10–11 sensors. For each cross‐validation fold, one partition is used as a test data set and all other folds are used as training data. For each algorithm, the training data are source reconstructed to gain an estimate of source dynamics. This source‐reconstructed activity is then forward mapped to the left out test sensors, and the temporal variance of the data recorded at these sensors explained by the brain activity is calculated as squared (temporal) correlation. The $r_{\mathrm{CV}}^{2}$ measure reported here is the average variance explained at each test sensor, averaged over folds. For visualization, we demonstrate a single test sensor. The same methods are repeated for empty‐room recordings, and we use ratio of variance explained in resting‐state versus empty room recordings as our measure of interest. (b,c) Resolution analysis. (b) For a seed voxel, the corresponding column of the resolution matrix (the point spread function; PSF) relates unit “true” activity at that voxel to source estimates at each voxel. The distance between the seed voxel and the voxel at the peak of the PSF is the PAD. Shown is an example PSF for a seed voxel at the red diamond. The arrow points from the seed to the peak of the PSF, and the length of the arrow is PAD. The SEPS is the PSF‐weighted sum of distances to each voxel from the seed. (c) Rows of the resolution matrix (the cross talk function; CTF) quantifies the influence of “true” activity at all voxels on the source estimate of the seed voxel. The SECT is the CTF analogue of SEPS. Here we show an example CTF for a seed voxel at the red diamond. In b,c, figures are plotted on the smoothed cortical surface for visualization purposes only. (d) External noise analysis. To test robustness to external noise, white noise is added to the MEG sensor‐level data. Correlation between source‐space solutions with and without added sensor noise is computed. The level of reduction in correlation as the sensor‐level noise increased quantifies an algorithm's sensitivity to external noise

We used two key analyses to compare source reconstruction algorithms (Figure 1). In the variance explained analysis, we estimated the variance of the empirical data explained by the source reconstruction. In the resolution analysis, we calculated measures based upon the resolution matrix (Hauk et al., 2011), which theoretically relates the activity based at dipole i to the estimate at dipole j.

#### 2.2.1 Variance explained analysis

A source reconstruction algorithm should accurately estimate the time course of the brain activity underpinning the M/EEG data. Therefore forward modeling to a new sensor should accurately predict the time series recorded at that sensor. Here, we use a 27‐fold cross‐validation procedure to assess source reconstruction solutions based on this assumption. The $N_{x}=274$ MEG sensors are randomly partitioned into 27 groups of 10 or 11 sensors using Matlab's *cvpartition* function. In each fold of cross validation, one of the 27 partitions are left out as a test set, and the remaining 26 partitions are used as training data. For a given individual, the same folds are used for all source‐reconstruction algorithms. The training data is source reconstructed to estimate brain activity $\hat{s}$ (Equation 2). The predicted data at each sensor in the test set is then estimated as ${\hat{x}}_{\text{test}}=L_{\text{test}}\hat{s}$, where $L_{\text{test}}$ is the row of the leadfield matrix corresponding to the test sensor. We quantify the squared Pearson correlation coefficient between ${\hat{x}}_{\text{test}}$ and the empirical data recorded by the test sensor, averaged over all test sensors and all cross validation folds, that is,

(3) $$r_{\mathrm{CV}}^{2}=\frac{1}{N_{x}}\sum_{\text{fold}=1}^{27}\sum_{\text{test}\in \text{fold}}{\left|\text{corr}\left(x_{\text{test}},{\hat{x}}_{\text{test}}\right)\right|}^{2}.$$

The squared Pearson correlation coefficient is the coefficient of determination of a linear model (Devore, 2012), that is, $r_{\mathrm{CV}}^{2}$ is the temporal variance of the empirical MEG time series explained by a linear regression with the predicted MEG time series from forward mapping the source solution.

Although quantities similar to $r_{\mathrm{CV}}^{2}$ has been used elsewhere (Little et al., 2018), as an isolated measure in the context of empirical data, a high $r_{\mathrm{CV}}^{2}$ value do not necessarily imply a good quality of source reconstruction. MEG recordings contain external noise and artifacts not due to brain signals, and hence an algorithm with $r_{\mathrm{CV}}^{2}=1$ is likely to be overfitting these phenomena. Considering $r_{\mathrm{CV}}^{2}$ alone would also result in unfair comparisons between the LSMN type estimates and beamformers. LSMN‐based algorithms minimize the L2‐norm of $\hat{x}-x$, and hence they can be more prone to overfitting and potentially have higher $r_{\mathrm{CV}}^{2}$ than beamforming counterparts. To resolve these issues, we additionally calculate cross‐validated $r^{2}$ for empty room data (here called $r_{\mathrm{ER}}^{2}$). An algorithm which has high $r_{\mathrm{ER}}^{2}$ is overfitting nonbrain signals. We therefore use the ratio $r_{\mathrm{CV}}^{2}/r_{\mathrm{ER}}^{2}$ as our measure of interest, which denotes $r_{\mathrm{CV}}^{2}$ normalized by the extent to which the algorithm overfits nonbrain signals.

It should also be noted that sLORETA and UNGMV normalize the inverse filters (Appendix A), meaning that the estimated source distributions are no longer in units of current density. Therefore, the forward mapping of the source estimate (${\hat{x}}_{\text{test}}$) will no longer be in the same units as the original MEG data ($x_{\text{test}}$). Since the correlation coefficient is independent of amplitude scalings, it is still valid to calculate $r_{\mathrm{CV}}^{2}$ (or $r_{\mathrm{ER}}^{2}$) despite these differences in units. In this regard, the variance explained statistic can be seen as quantifying the extent to which the temporal fluctuations in source reconstructed solutions explain the temporal fluctuations at test sensors.

#### 2.2.2 Resolution analysis

We further used three measures derived from the resolution matrix (Hauk et al., 2011) to compare algorithms: the peak activation displacement (PAD), spatial extent of cross talk (SECT) and spatial extent of point spread (SEPS). While resolution metrics quantify the performance of each source in isolation, for linear methods they have also been recommended as appropriate for studying distributed source activity (Hauk et al., 2011; Hauk et al., 2019; Hedrich et al., 2017).

Substituting Equation (1) into Equation (2), we obtain

(4) $$\hat{s}=\Phi \mathrm{Ls}+\Phi \eta .$$

Although we cannot know the true source dynamics s, we know they are related to the estimated source dynamics $\hat{s}$ by the equation

(5) $$\hat{s}=\mathrm{Rs}+\bar{\eta },$$

where R=ΦL ($R\in {\mathbb{R}}^{N_{s}\times N_{s}}$) is the *resolution matrix* and $\bar{\eta }=\Phi \eta$ is a noise term (specifically, it is the source space projection of the measurement noise). The i'th column of R is called the point spread function of dipole i (${\mathrm{PSF}}_{i}$), which corresponds to the influence of activity at dipole i on the estimate at all dipoles in the source reconstruction.

The PAD of dipole i (${\mathrm{PAD}}_{i}$) (Hauk et al., 2019) is thus given by

(6) $${\mathrm{PAD}}_{i}=∥r\left[i\right]-r[\underset{j}{\arg\;\max}{\mathrm{PSF}}_{i}\left(j\right)]∥,$$

where $r\left[k\right]$ is the location of dipole k. That is, a dipole i located at $r\left[i\right]$ most strongly influences the estimate of dipole j at location $r\left[j\right]$, and PAD is the Euclidean distance between these two dipoles. Previous literature has called this metric the “localization error,” but since the aim of the study is not to localize a particular source, this term is potentially misleading, as the metric is interpreted in a slightly different manner to those used in typical localization studies. A more detailed discussion of this point and interpretation of the measure can be found in Section 4.3.

While PAD estimates the distance between a source origin and its peak in the estimated source data, it does not quantify how sharp or blurred this estimate is, that is, the spatial extent of leakage dispersing from the source. We quantify this leakage using a metric previously called “spatial dispersion (SD)” (Hauk et al., 2019), but here called the SEPS to avoid misinterpretation (see Section 4.3 for a justification of this difference in naming convention). The SEPS from dipole i is calculated as

(7) $${\text{SEPS}}_{i}=\sqrt{\frac{\sum_{j}R_{\mathrm{ji}}^{2}∥r\left[j\right]-r\left[i\right]{∥}^{2}}{\sum_{j}R_{\mathrm{ji}}^{2}}},$$

where $R_{\mathrm{ji}}={\mathrm{PSF}}_{i}\left(j\right)$ is element $\left(j,i\right)$ of R. In the idealized solution with no leakage, R=I. As nondiagonals become nonzero, so does SEPS.

The point spread function quantifies the leakage at other dipoles as a result of activity at i. Conversely, the cross talk function of dipole i (${\mathrm{CTF}}_{i}$) quantifies the leakage at dipole i due to activity at other dipoles. ${\mathrm{CTF}}_{i}$ is the i'th row of R. We quantify the spatial extent of dipoles whose leakage influences the estimate at i using the spatial extent of cross talk (${\text{SECT}}_{i}$),

(8) $${\text{SECT}}_{i}=\sqrt{\frac{\sum_{j}R_{\mathrm{ij}}^{2}∥r\left[j\right]-r\left[i\right]{∥}^{2}}{\sum_{j}R_{\mathrm{ij}}^{2}}},$$

which is the CTF counterpart to SEPS for the point spread function. Here, $R_{\mathrm{ji}}={\mathrm{CTF}}_{i}\left(j\right)$ is element $\left(i,j\right)$ of R. For all resolution metrics, values reported are averaged over all dipoles.

#### 2.2.3 Comparing source reconstruction algorithms using parcellated data

We extended the comparisons between source reconstruction algorithms to parcellated data. For the variance explained analysis, cortical parcellation was performed by taking the first principal component of all voxels within a ROI (Tait et al., 2019) to obtain a single time course for the ROI. The representative time course of the ROI was then mapped back to voxel space using the column of the PCA mixing matrix corresponding to the first principal component. The variance explained analysis with cross‐validation was then performed as described in Section 2.2.1. For the resolution analysis, we defined fractional PAD for ROI ω (${\text{fPAD}}_{\omega }$) as the fraction of dipoles within ω that had peaks of the PSF outside of ω. As parcellated counterparts to SEPS and SECT, we calculated the zero‐lag correlation between time courses of anatomically neighboring ROIs, since highly correlated neighboring regions suggests large spatial leakage. This was averaged over all pairs of anatomical neighbors, to obtain the mean neighbor correlation (mNC).

#### 2.2.4 Statistical analysis

For all measures, values reported were averaged over all dipoles or ROIs unless otherwise stated. Statistical analysis of metrics across algorithms required tests accounting for repeated measures to address the within‐subject experimental design. Friedman tests were used for group level analyses, and pairwise comparisons were performed using the Wilcoxon signed rank test. All p‐values reported for pairwise comparisons were false discovery rate corrected using the Benjamini–Hochberg procedure.

### 2.3 Reducing the HCP‐MMP atlas

We proposed a data‐driven approach to reduce the HCP‐MMP atlas from 360 to 230 cortical ROIs. This number was chosen such that the number of ROIs was less than or equal to the rank of our preprocessed MEG data (minimum rank 233). This was motivated by the fact it is commonplace to orthogonalize parcellated data, for example, for functional connectivity analyses, and therefore by using fewer ROIs than the rank of the data we avoid rank deficiency.

Glasser et al. (2016) grouped 180 cortical ROIs per hemisphere into 22 anatomical “clusters” of regions, and our reduced atlas aimed to maintain these clusters. Consider a cluster Ω. For a given ROI ω∈Ω, the influence of that ROI over the MEG was defined as

(9) $$∥L_{\omega }∥=\sum_{j\in \omega }∥L_{j}∥,$$

where $L_{j}$ is the column of the leadfield matrix corresponding to dipole j. Here, we take the average value of $∥L_{\omega }∥$ over subjects using our MRI derived leadfields. This average is demonstrated to be representative of all participants in Supplementary Figure S2. Then the corresponding influence of Ω is calculated as

(10) $$∥L_{\Omega }∥=\sum_{\omega \in \Omega }∥L_{\omega }∥.$$

The number of ROIs in Ω should ideally be proportional to $∥L_{\Omega }∥$. Then, given a target number of ROIs per hemisphere (here chosen to be 125), a simple algorithm was used to determine the optimal number of ROIs for each cluster, such that the target total is achieved. The algorithm is described in detail in Appendix B. Subsequently, we used the detailed neuroanatomical results presented by Glasser et al. (2016) to merge neighboring ROIs within a cluster that had similar neuroanatomical attributes (prioritizing resting‐state functional connectivity) until the cluster had the optimal number of ROIs and a uniform distribution of ROI strengths. A full list of ROIs and descriptions of which ROIs were merged is given in Supporting Information.

### 2.4 Participants

Eleven healthy participants (8 female, 3 male) were recruited from Cardiff University School of Psychology participant panel (age range 19–24 years, mean age 20.45 years). All participants had normal or corrected‐to‐normal vision, and none reported a history of neurological or psychiatric illness. Written consent was obtained from all participants. The study was approved by the Cardiff University School of Psychology Research Ethics Committee.

### 2.5 MEG and MRI data acquisition

Whole‐head resting‐state MEG recordings were made using a 275‐channel CTF radial gradiometer system (CTF Systems, Canada) at a sampling rate of 1,200 Hz. An additional 29 reference channels were recorded for noise cancellation purposes and the primary sensors were analyzed as synthetic third‐order gradiometers (Vrba & Robinson, 2001). One sensor was turned off during recording due to excessive sensor noise (i.e., $N_{x}=274$ gradiometers). Subjects were instructed to sit comfortably in the MEG chair while their head was supported with a chin rest and with eyes open focus on a fixation point on a grey background. Horizontal and vertical electro‐oculograms (EOG) were recorded to monitor blinks and eye movements. The horizontal electrodes were placed on temples, and vertical ones, above and below the eye. For MEG/MRI co‐registration, the head shape with the position of the coils was digitized using a Polhemus FASTRAK (Colchester, Vermont). Each recording session lasted approximately eight minutes.

All participants also underwent a whole‐brain MRI scan on a Siemens 3T Connectom MRI scanner and a 32‐channel receiver head coil (Siemens Medical Systems). We used a T1‐weighted magnetization prepared rapid gradient echo sequence (MPRAGE; echo time: 3.06 ms; repetition time: 2,250 ms sequence, flip angle: 9°, field‐of‐view: = 256 × 256 mm, acquisition matrix: 256 × 256, voxel size: 1 × 1 × 1 mm).

### 2.6 Data preprocessing and forward modeling

Continuous raw MEG data was imported to Fieldtrip (Oostenveld et al., 2011), bandpass filtered at 1–100 Hz (fourth order two‐pass Butterworth filter) and subsequently notch filtered at 50 and 100 Hz to remove line noise. Visual and cardiac artifacts were removed using ICA decomposition, using the “fastica” algorithm (Hyvärinen, 1999). Identification of visual artifacts was aided by simultaneous EOG recordings. Between 2 and 5 components were removed for each subject. Data were then downsampled to 256 Hz and a randomly selected 30 second epoch was chosen. The Fieldtrip functions “ft_artifact_jump” and “ft_artifact_clip” were used to validate that this epoch was free from clip and jump artifacts.

From T1‐weighted MRI image, extraction of the inner skull, scalp, pial, and grey matter/white matter boundary surfaces was performed with the Freesurfer (Dale, Fischl, & Sereno, 1999, http://surfer.nmr.mgh.harvard.edu). These surfaces and the MRI scan were imported into the Brainstorm software (Tadel, Baillet, Mosher, Pantazis, & Leahy, 2011) and an automated procedure used to align these data to the MNI coordinate system. The midpoint between the pial surface and grey matter/white matter boundary was extracted and downsampled to 10,000 homogeneously spaced vertices (median distance between vertices 4.32 mm; Figure S3) to generate a cortical surface of dipole locations using the “iso2mesh” software (Fang & Boas, 2009) implemented in Brainstorm. The inner skull surface was similarly downsampled to 500 vertices. These surfaces were then exported to Matlab, where the scalp surface was used to align the structural data with the MEG digitizers. The aligned MEG gradiometers, inner skull surface, and cortical surface were then used to construct a realistic, subject specific, single shell forward model (Nolte, 2003). Unless stated otherwise, dipole orientations were fixed normal to the cortical surface (Dale et al., 2000; Hillebrand & Barnes, 2003) under the assumption that M/EEG signals are primarily generated by the postsynaptic currents in the dendrites of large vertically oriented pyramidal neurons in layers III, V, and VI of the cortex (Olejniczak, 2006).

For the parcellation analysis, the cortical surface was further aligned to the Human Connectome Project Multimodal Parcelation (HCP‐MMP) atlas (179 cortical ROIs per hemisphere, excluding the hippocampus; Glasser et al. (2016)) in Freesurfer, and Matlab was used to merge ROIs according to the parcellation reduction procedure of Section 2.3.

## 3 RESULTS

### 3.1 Comparing source reconstruction algorithms

**FIGURE 2:**
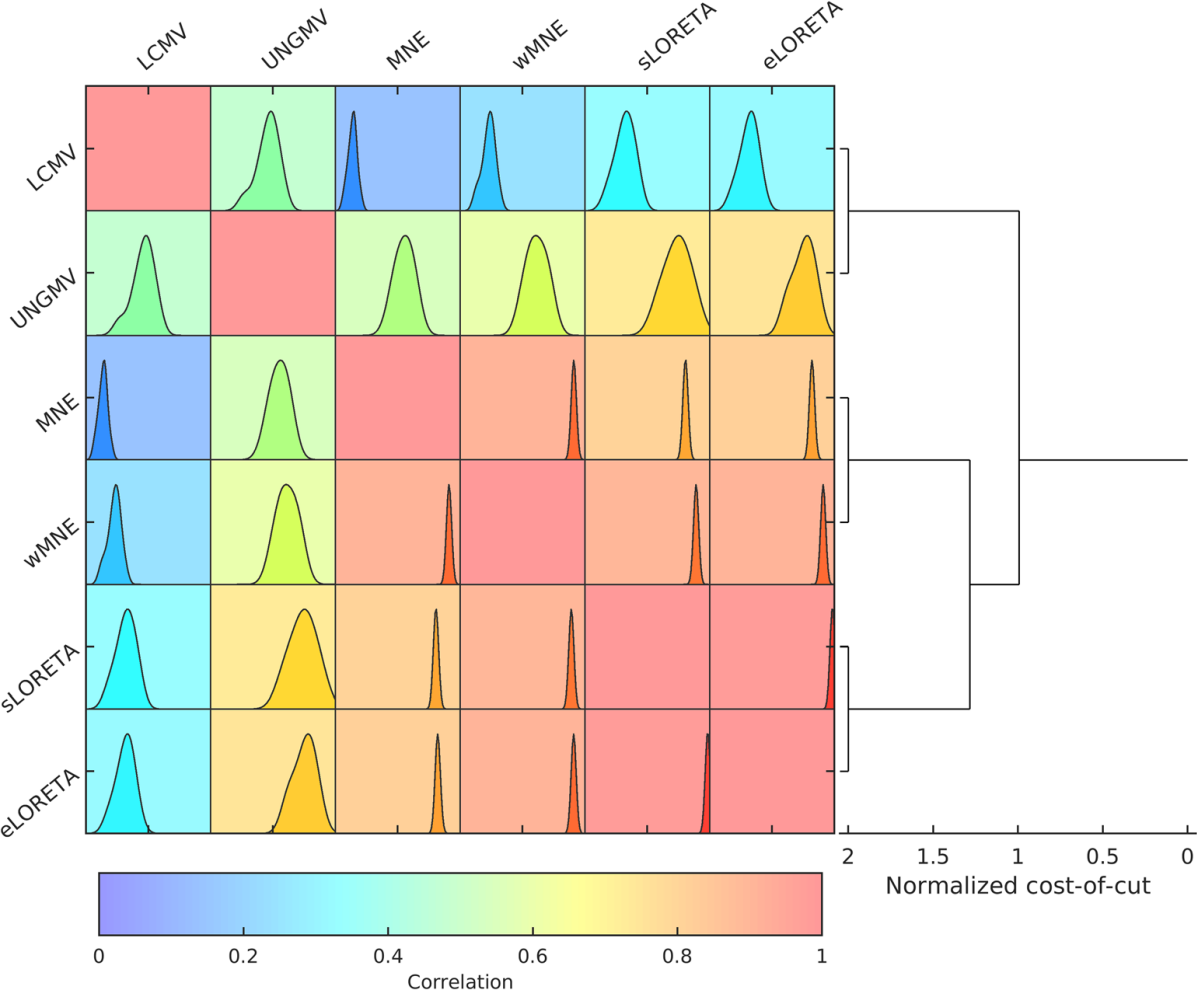
Correlation between different source reconstruction algorithms. Each algorithm was used to source reconstruct the MEG data, and correlation in the source space solutions of each pair of algorithms was calculated. For each pair of algorithms, the background color of the correlation matrix represents median value of correlation over all participants, while the inlaid plot shows the distribution over all subjects (the *x*‐axis is calibrated evenly for all plots, in the range [0,1]). No negative correlations were found. Hierarchical clustering of the median network is shown on the right to group algorithms based on similarity. Clustering used a spectral normalized cuts algorithm (Shi & Malik, 2000), and the *x*‐axis shows the cost of the cut. Therefore clusters with higher cost‐of‐cut are more strongly clustered, that is, contain higher correlations between algorithms

Figure 2 shows the correlation between the source space solution of pairs of algorithms, and hierarchical clustering of the algorithms based on these correlations. As expected, source reconstruction solutions differed to various extents between different algorithms. eLORETA and sLORETA were highly correlated, which is in line with past comparisons between these algorithms (Jatoi, Jamel, Malik, & Faye, 2014). There was also a strong correlation between MNE and wMNE solutions. More broadly, these four LSMN algorithms formed a larger cluster, with correlations of at least ∼0.7 between all pairs of algorithms. The two beamformers had a correlation of approximately .5 with each other, forming an additional cluster. The LCMV solution had low correlations with the LSMN cluster, while UNGMV had a stronger correlation with algorithms in this cluster, particularly eLORETA and sLORETA.

#### 3.1.1 Variance explained analysis

**FIGURE 3:**
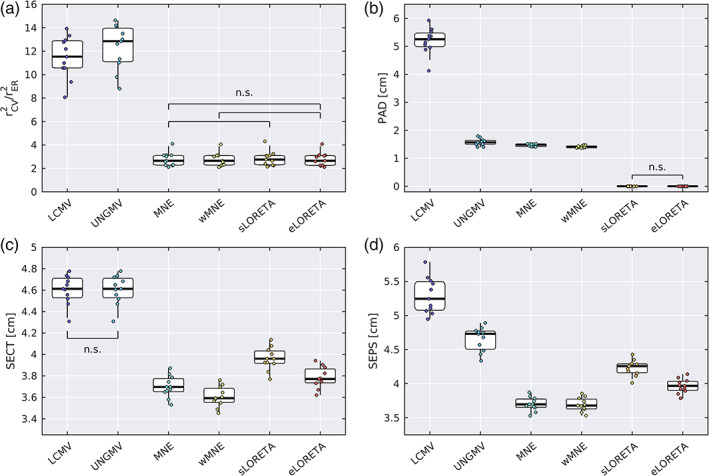
Statistics from variance explained and resolution analysis. (a) Ratio of cross‐validated variance explained in empirical data and empty room recordings ($r_{\mathrm{CV}}^{2}/r_{\mathrm{ER}}^{2}$) for each source reconstruction algorithm. (b) Peak activity displacement (PAD). (c) Spatial extent of cross talk (SECT). (d) Spatial extent of point spread (SEPS). For all figures, group‐wise analyses demonstrated a significant effect of algorithms. Pairwise analyses identified significant differences for all pairs except those marked as nonsignificant (n.s.), following false discovery rate correction. In the box plots, the range (whiskers), interquartile range (boxes), median (horizontal line), are shown, with values for each participant marked by dots to the left

Performance of the algorithms was first quantified using an analysis of variance of empirical data explained by the source reconstruction as a ratio of the same measure in empty room recordings, $r_{\mathrm{CV}}^{2}/r_{\mathrm{ER}}^{2}$ (Equation 3 and see Section 2.2). The values of $r_{\mathrm{CV}}^{2}/r_{\mathrm{ER}}^{2}$ are shown for each algorithm in Figure 3a, quantifying the temporal variance of sensor data explained by the source reconstructed data, normalized by ambient noise (empty‐room recording). There was a significant main effect of algorithm on $r_{\mathrm{CV}}^{2}/r_{\mathrm{ER}}^{2}$ ($\chi^{2}=47.26$, $p=5.09\times 10^{-9}$, Friedman test), and pairwise comparisons demonstrated significant differences between all pairs of algorithms except MNE versus sLORETA, MNE versus eLORETA, and wMNE versus eLORETA. Means, standard errors, and post‐hoc pairwise p‐values are reported in Table S1. The beamformers notably outperformed the LSMN methods, with $r_{\mathrm{CV}}^{2}/r_{\mathrm{ER}}^{2}=12.354\pm 0.571$ (UNGMV) and 11.480±0.536 (LCMV). sLORETA was highest performing of the LSMN methods with $r_{\mathrm{CV}}^{2}/r_{\mathrm{ER}}^{2}=2.840\pm 0.189$. This was followed by MNE, then wMNE and eLORETA which performed comparably (p=.8311).

To examine the generalizability of these results, we varied consistently the regularization parameter across of all algorithms by changing their predicted SNRs (from −10 to 10 dB, see Appendix A.3). The relative ranking of $r_{\mathrm{CV}}^{2}/r_{\mathrm{ER}}^{2}$ between algorithms maintained across SNRs, albeit beamformers showed much larger $r_{\mathrm{CV}}^{2}/r_{\mathrm{ER}}^{2}$ values than LSMN algorithms at high SNR levels (Figure S1a). To explore this result further, Figure S4 shows the $r_{\mathrm{CV}}^{2}$ and $r_{\mathrm{ER}}^{2}$ values separately. Interestingly, the four LSMN algorithms explain more of the resting‐state data ($r_{\mathrm{CV}}^{2}$ between 81.4 and 93.6%) than the beamformers (between 55.0 and 75.7%). However, the LSMN algorithms also explain a large percentage of the empty‐room data ($r_{\mathrm{ER}}^{2}$ 19.3–41.2%) compared to the beamformers ($r_{\mathrm{ER}}^{2}$ 4.6–7.6%) because the latter are less affected by noise outside the brain (Hincapié et al., 2017; Van Veen et al., 1997). This explains the larger ratio $r_{\mathrm{CV}}^{2}/r_{\mathrm{ER}}^{2}$ in the beamformers than the LSMN type estimates.

#### 3.1.2 Resolution analysis

In the first resolution analysis, we quantified the peak activity displacement (PAD) for each algorithm (Equation 6). There was a significant main effect of algorithm on PAD (Figure 3b, $\chi^{2}=52.75$, $p=3.78\times 10^{-10}$), with significant differences between all pairs of algorithms except for comparisons between sLORETA and eLORETA. As previously reported numerically (Hauk et al., 2019; Pascual‐Marqui et al., 2018) and theoretically expected (Pascual‐Marqui, 2007), eLORETA and sLORETA had zero PAD. wMNE had the next lowest PAD, followed by MNE and UNGMV, while LCMV had the notably the largest PAD.

We also compared the SEPS (Equation 7) and cross talk (SECT; Equation 8) influencing the source estimates. There was a significant main effect of algorithm on both SECT (Figure 3c, $\chi^{2}=54.18$, $p=1.92\times 10^{-10}$) and SEPS (Figure 3d, $\chi^{2}=54.48$, $p=1.67\times 10^{-10}$), with significant differences between all pairs of algorithms (except for LCMV vs. UNGMV for SECT). For both measures, the ranking of algorithms was the same. wMNE had the lowest SECT/SEPS, followed by MNE, eLORETA, and sLORETA. The algorithms with largest SECT/SEPS were the beamformers; UNGMV had lower SEPS than LCMV, whereas they had identical values of SECT since setting columns of the spatial filter to unit norm has no effect on the cross talk function.

Means, standard errors, and post‐hoc pairwise p‐values for all resolution metrics are reported in Tables S2–S4. Results of the resolution analyses for a range of predicted SNRs are shown in Figure S1b–d.

#### 3.1.3 Dipole orientations

The results above were obtained from forward models with anatomical dipole orientations that are normal to the cortical surface (Figure S5). In Figure S6, we showed that the relative ranking of algorithms is maintained in most measures (i.e., $r_{\mathrm{CV}}^{2}/r_{\mathrm{ER}}^{2}$, PAD, SEPS, and SECT) if using eigendecomposition to define dipole orientation (Sekihara, Nagarajan, Peoppel, & Marantz, 2004; Supplementary Figure S5), suggesting that our results do not depend on anatomical orientation constraints. However, it should be noted that all algorithms had significantly lower SECT and SEPS, in the case of eigendecomposition dipole orientations. PAD was also significantly lower for LCMV, UNGMV, and wMNE when eigendecomposition was used (however, significantly higher for MNE). Therefore, resolution metrics largely performed better in the case of eigendecomposition. Conversely, $r_{\mathrm{CV}}^{2}/r_{\mathrm{ER}}^{2}$ on average significantly decreased when eigendecomposition was used for the beamformers, suggesting worse performance on this measure when eigendecomposition was used.

#### 3.1.4 Effects of external white noise

A key factor affecting the source reconstruction is SNR. An ideal source reconstruction algorithm should source reconstruct brain data while rejecting nonbrain signals such as external noise. In practice, this cannot be perfectly achieved by any source reconstruction algorithm. Yet, different algorithms differ in the degree to which the source‐space solution is influenced by nonbrain noise. In Figure 5, we compared the sensitivity of source reconstruction algorithms to the addition of artificial external white noise. Artificial Gaussian i.i.d. sensor noise was added with variance $\sigma^{2}\cdot \text{trace}\left(C_{x}\right)/N_{x}$, where $C_{x}$ is the sensor covariance matrix and $N_{x}$ is the number of sensors. The regularization parameter was appropriately adjusted to account for the new predicted SNR, and the noisy data was source reconstructed (without cross‐validation). To quantify the degree to which source space solutions were altered by the addition of sensor noise, we calculated spatiotemporal correlation between the source‐space solutions with and without artificial sensor noise (Figure 5a).

An ideal algorithm will only source reconstruct the brain signal and ignore the noise, and so this correlation should remain at one. In practice, as the variance of the artificial sensor noise is increased, all algorithms see a drop in source space correlation (i.e., the source solution changes due to the artificially added sensor noise). We calculated the standard deviation of correlation scores across different levels of noise as a single measure of the degree to which external noise influences the solution for a given algorithm (Figure 5b). There was a significant main effect of algorithm on this standard deviation metric ($p=3.16\times 10^{-9}$, Friedman test) and significant pairwise differences between all pairs of algorithms except UNGMV and eLORETA. Of the algorithms, MNE and wMNE performed notably the worst, being strongly effected by the addition of sensor noise.

### 3.2 The reduced HCP‐MMP atlas

We proposed a data‐driven method (see Section 2.3) to reduce the 360 ROIs in the HCP‐MMP atlas to 230 ROIs, fewer than the rank of our MEG data and hence appropriate for further analysis (Colclough et al., 2015). The essential criterion for ROI reduction is to merge neighboring ROIs that have small influences over MEG sensor‐level data (given by the MEG leadfield matrix, see Equation 10), while maintaining all the clusters in the original HCP‐MMP atlas with similar microstructural, functional and connectivity profiles (Glasser et al., 2016). As such, the reduced HCP‐MMP atlas is optimized for MEG source reconstruction.

**FIGURE 4:**
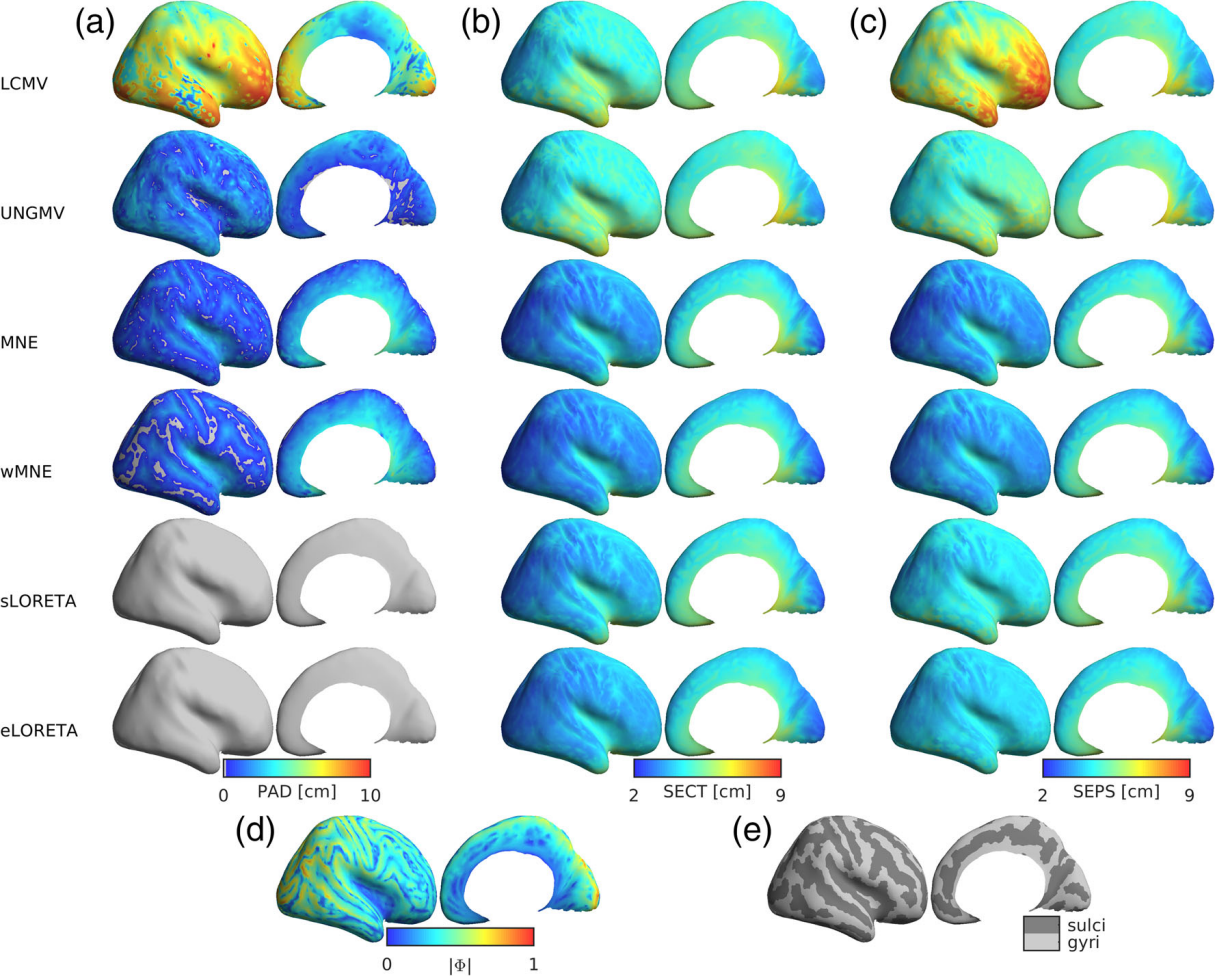
Spatial distributions of resolution metrics. (a–c) Lateral and medial views (left and right columns) of PAD, SECT, and SEPS (respectively) for each dipole in each algorithm. (d) The norm of the leadfield for each dipole. (e) Anatomical maps of gyri and sulci, derived in Freesurfer. For all figures, distributions are shown on a smoothed cortical surface for visualization purposes, and are shown for the same representative participant

The reduced atlas is shown in Figure 6, and a detailed description of all ROI reductions to the HCP‐MMP atlas is given in Supporting Information. Merged ROIs were found mostly in the less superficial areas such as medial temporal regions (including the ventral visual stream and medial temporal cortex), the lateral sulcus (including early auditory, insular, and opercular cortices), and medial regions (including the cingulate, medial prefrontal, and orbitofrontal cortices; Figure 6c,f). By merging ROIs in these regions that have small influences over the MEG, the distributions of influence of ROIs became more homogeneous (Figure 6a,d). For example, the insular cortex in the original atlas consisted of thirteen small and deep ROIs per hemisphere, which had little influence on the MEG (Figure 6a–c). Our algorithm determined the optimum number of regions was three, so by merging insula ROIs which were anatomical neighbors and had similar resting‐state functional connectivity as reported by Glasser et al. (2016), we achieved three larger ROIs with comparable influence over the MEG to ROIs in more superficial regions (Figure 6d–f). These merges can be justified by our resolution analysis (Figure 4b), which demonstrates that the insular had much lower resolution (i.e., higher SECT/SEPS) than superficial regions for all source reconstruction algorithms.

The reduced HCP‐MMP atlas was further validated in a larger sample including an additional 20 participants. For results in the larger sample, see Figure S7.

### 3.3 Comparing source reconstruction algorithms using parcellated data

Based on the reduced HCP‐MMP atlas Figure 6e, we parcellated all voxelwise solutions of source reconstruction algorithms into 230 ROIs. For each ROI, its representative time course was obtained from the first principal component across all voxels within that ROI. We then compared the source reconstruction algorithms using the parcellated, representative time courses of ROIs. The variance explained analysis and resolution metrics were adapted accordingly for parcellated data (see Section 2.2.3).

The values of cross‐validated variance explained ($r_{\mathrm{CV}}^{2}/r_{\mathrm{ER}}^{2}$) are shown for each algorithm in Figure 7a. There was a significant main effect of algorithm (Figure 7a, $\chi^{2}=52.82$, $p=3.67\times 10^{-10}$), and pairwise comparisons demonstrated significant differences between all pairs of algorithms except MNE versus wMNE. Means, errors, and p‐values for the pairwise comparisons are given in Table S5. Similar to the unparcellated data, UNGMV and LCMV had the largest $r_{\mathrm{CV}}^{2}/r_{\mathrm{ER}}^{2}$. For the LSMN methods, wMNE/MNE were the best performing, followed by sLORETA and finally eLORETA. All algorithms had a parcellated $r_{\mathrm{CV}}^{2}$ value lower than that of the voxel‐wise $r_{\mathrm{CV}}^{2}$, which is expected because the PCA‐based representative time course do not contain all the information in an ROI.

Fractional peak activity displacement (fPAD) was calculated as a parcellation based counterpart to PAD (Figure 7c). By definition, zero PAD results in zero fPAD, so consistent with unparcellated results we found zero fPAD for sLORETA and eLORETA. There was a significant effect of algorithm on fPAD ($\chi^{2}=55$, $p=1.31\times 10^{-10}$), with significant differences in all tests except those between algorithms with zero fPAD. Consistent with the PAD in unparcellated results, wMNE had the lowest nonzero fPAD, followed by MNE, then UNGMV. LCMV had fPAD of 98.0±0.3%, suggesting that the majority of source reconstructed signal is maximally attributed to a wrong ROI.

Finally, mNC was used as a measure of resolution/leakage of the parcellated data, a counterpart of the SEPS/SECT resolution measures for voxelwise analyses (Figure 7d). Low mNC is suggestive of low leakage, since resolution is high enough to distinguish activity at neighboring ROIs. There was a significant main effect of algorithm on mNC ($\chi^{2}=42.12$, $p=5.58\times 10^{-8}$). LCMV, MNE, and wMNE had the lowest mNC values, with no significant difference between these algorithms. All three algorithms were significantly lower than the remaining algorithms. UNGMV and eLORETA were the next lowest mNC, with no significant difference between these algorithms. Finally, sLORETA had significantly higher mNC than all other algorithms.

Means, standard errors, and post‐hoc pairwise p‐values for all parcellated metrics are reported in Tables S5–S7.

## 4 DISCUSSION

The current study consisted of two main aims, unified by the goal of developing a methodology to guide future source reconstruction analyses of resting‐state MEG. First, we systemically assessed which of the many inverse algorithms for source reconstructing MEG data was most suitable for use with empirical resting‐state data. This was achieved through the measures of variance explained (specifically, the ratio of cross‐validated temporal variance explained in resting‐state vs. empty room data) and resolution metrics. Second, we presented a reduced atlas that is based on the high resolution, multi‐modal parcellation of the Human Connectome Project (Glasser et al., 2016), which is optimized for use with resting‐state MEG data. The main contributions and implications of our results are discussed in the following sections.

### 4.1 Comparison of source localization algorithms

Our comparisons and recommendations of source localization algorithms are summarized in Table 1. There is no single algorithm, which is an all‐purpose first choice, with each algorithm demonstrating strengths and weaknesses. For example, we found the primary advantage to the beamformers over the LSMN algorithms were minimal influence from external sources, evidenced by having the lowest $r_{\mathrm{ER}}^{2}$ values (and highest gain in temporal variance explained vs. resting‐state, $r_{\mathrm{CV}}^{2}/r_{\mathrm{ER}}^{2}$) in voxel‐wise data and small changes in source space solutions as sensor noise is added. These results are suggestive that the beamformed source space solutions are predominantly influenced by the macroscopic neural dynamics and not external noise. The distinct difference between the two categories of algorithms is to be expected. Beamformers are designed to minimize variance from sources outside of the dipole of interest (Van Veen et al., 1997), while LSMN algorithms are designed to explain the sensor data (Fuchs et al., 1999), including external influences. Point dipole simulations have also verified that beamformers are less sensitive to external noise than minimum norm estimates (Hincapié et al., 2017). However, we found beamforming comes at the cost of higher PAD (in line with past studies [Pascual‐Marqui et al., 2018; Halder et al., 2019; Hauk et al., 2019]) and lower spatial resolution than the LSMN algorithms.

**TABLE 1:** Summary of results and recommended use of algorithms

	High temporal variance explained (ratio)		Zero peak activity displacement		Low leakage		Robust to additional sensor noise	Recommended use
Algorithm	voxel	ROI	voxel	ROI	voxel	ROI		
LCMV	✓	✓	✗	✗	✗	✓	**✓**	UNGMV is usually preferred, except for when comparing power statistics between conditions.
UNGMV	✓	✓	✗	✗	✗	∼	✓	If data have low SNR/artifacts. Preferred beamformer, except for when comparing power statistics between conditions.
MNE	✗	✗	✗	✗	✓	✓	✗	wMNE is usually preferred.
wMNE	✗	✗	✗	✗	**✓**	✓	✗	For very clean (high SNR) data if minimizing leakage is a priority (at the cost of localization errors).
sLORETA	✗	✗	**✓**	**✓**	✓	✗	✓	If data has low SNR and a LSMN method is preferred over beamforming.
eLORETA	✗	✗	**✓**	**✓**	✓	∼	✓	If there is no strong prior knowledge on SNR and data is clean from artifacts, or strong requirements for highest spatial resolution. When parcellating using high‐resolution atlas.

*Note*: For all measures, which were analyzed at both the voxel and ROI level, subheadings “voxel” and “ROI” specify to which level is referred. Checks (✓) show highly performing algorithms, tildes (∼) show middling performing algorithms, crosses (✗) show poorly performing algorithms. Bold checks (**✓**) and crosses (✗) show the best and worst performing algorithms respectively for each column. For the “Zero peak activity displacement” columns, well performing algorithms were those with zero PAD/fPAD, while poor performing algorithms were those with nonzero PAD/fPAD. For all other columns, we binned the mean score of each algorithm (across participants) into three evenly spaced bins, each with a width of 1/3 of the range across the algorithms. SECT and SEPS are summarized together in the “Low leakage (voxel)” column, due to consistent results between the measures. More detailed discussion of conclusions reached in the “Recommended use” column is given in Section 4.1.

For analyses using data parcellated into ROIs, our findings suggest eLORETA is a useful tool. In line with theoretical results (Pascual‐Marqui, 2007) and simulated dipoles (Pascual‐Marqui et al., 2018), we found both eLORETA and sLORETA had zero PAD, resulting in source activity at a dipole in ROI ω always having peak estimated activity in ω, that is, zero fPAD. Conversely, the remaining algorithms had nonzero PAD, and on the parcellated level we found between 65% (wMNE) and 98% (LCMV) of dipoles had peak activation outside of their correct ROIs (Figure 7c), meaning activity is largely attributed to the wrong ROIs. Since eLORETA had significantly better resolution properties than sLORETA at both the voxel (Figures 3c,d and 4b,c) and ROI (Figure 7d) levels, and explained more data than sLORETA in the parcellated paradigm (Figure 7a, Table S5), our results suggest the use of eLORETA for distributed imaging and subsequent parcellation into ROIs. Using a different methodology to the one presented here, Finger et al. (2016) found eLORETA outperformed other algorithms such as MNE and LCMV in ROI‐level (parcellated) resting‐state data. It should be noted, however, that the ROI level results presented here used a high resolution (230 ROI) atlas, and fPAD is likely to be reduced if a lower resolution atlas (i.e., the cortex is sectioned into fewer ROIs) such as the AAL Tzourio‐Mazoyer et al., 2002) is used. In this case, UNGMV or wMNE may also be appropriate. Furthermore, other approaches to working on the ROI level were not addressed here. A prime example would be placing a small number of “virtual electrodes” at centroids of ROIs (Engels et al., 2016; Hillebrand et al., 2016), in which case a beamformer would be preferred, since the beamformer solution at one dipole is not dependent on the rest of the source space (Van Veen et al., 1997).

**FIGURE 5:**
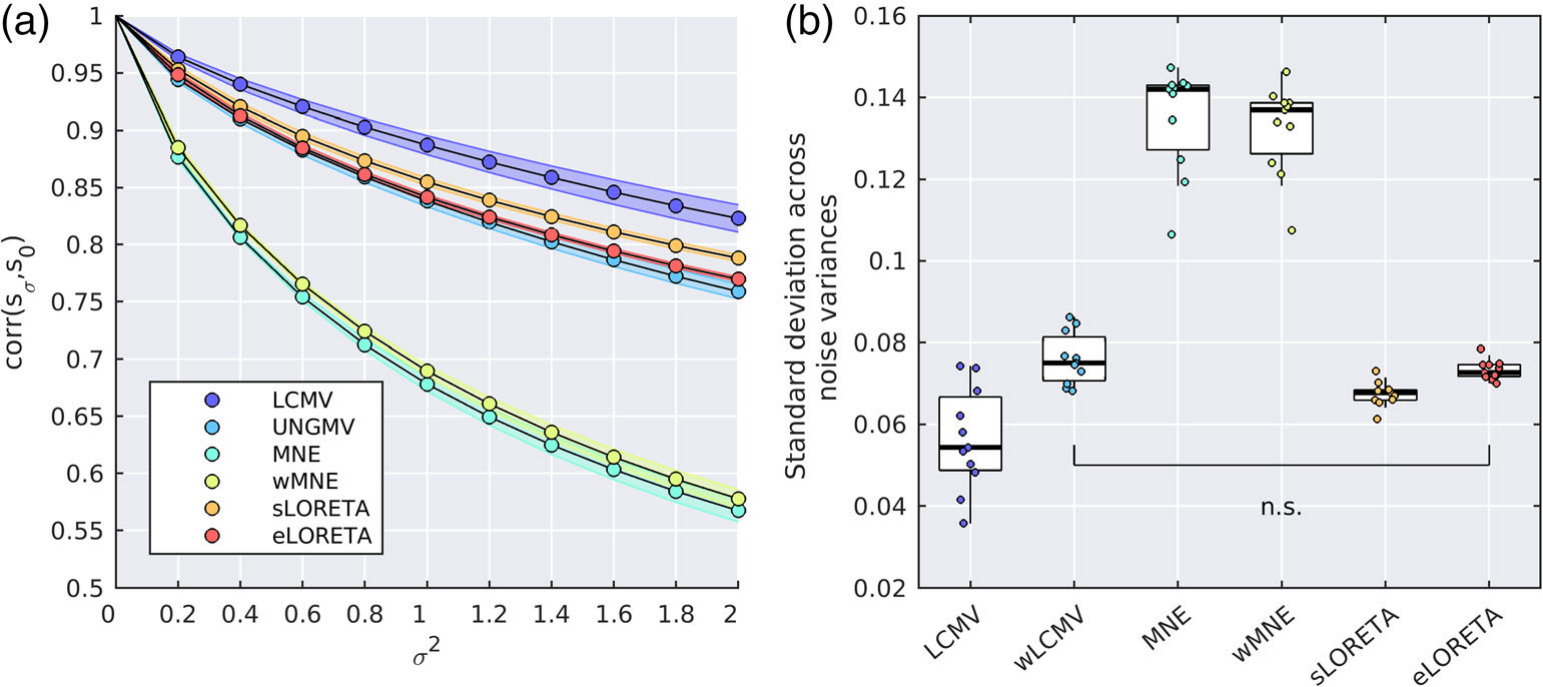
Correlation between source space solutions as sensor noise is added. Noise was added to the sensor data with variance $\sigma^{2}\cdot \text{trace}\left(C_{x}\right)/N_{x}$, where $C_{x}$ is the sensor data covariance matrix and $N_{x}$ is the number of sensors. We denote $s_{\sigma }$ as the source space solution to the noisy sensor data (without cross‐validation). The regularization parameter was adjusted according to the new predicted SNR due to added noise. (a) Correlation (averaged over dipoles) between $s_{\sigma }$ and $s_{0}$ (i.e., the source‐spaced solutions with and without noise added respectively) against noise variance $\sigma^{2}$. Shaded areas shown standard error of the mean. (b) The standard deviation of $\text{corr}\left(s_{\sigma },s_{0}\right)$ across noise levels, which (due to the monotonic decrease in correlation scores as noise increases) quantifies the extent to which an algorithm's solution changes as noise is artificially added. There was a significant difference between all pairs of algorithms except those marked nonsignificant (n.s.)

When working at the voxel‐level, there is no “one size fits all algorithm,” and the choice of algorithm is likely depends on the proposed analyses. If spatial accuracy is a priority—for example, localizing topographies of ICA derived resting‐state networks—our results suggest the use of eLORETA due to zero PAD and good performance on the leakage metrics (Figure 3). This is supported by the study of Liu et al. (2018), who found eLORETA outperformed algorithms such as MNE and LCMV in such an analysis. If minimizing leakage is the top priority, then wMNE had the lowest leakage metrics, but this comes at the cost of increased PAD and lowered robustness to noise. Conversely, if signal contamination from measurement noise or muscle artifacts is of concern, or the aim is to study the temporal evolution of the source dynamics and minimizing temporal fluctuations due to background activity is a priority, one may place spatial resolution properties at a lower priority than accurate temporal dynamics and limited noise contamination. In this case, our results suggest the use of a beamformer (for reasons described above). Alternatively, the optimal choice from the LSMN methods would be sLORETA due to having the highest $r_{\mathrm{CV}}^{2}/r_{\mathrm{ER}}^{2}$ of these methods (Figure 3a) and limited influence of external noise (Figure 5). While this study only considered sensor level white Gaussian noise, theoretically sLORETA will remain unbiased in the presence of arbitrarily structured measurement or biological noise (Pascual‐Marqui, 2007).

In the absence of a particularly strong priority for specifically minimizing either leakage or background activity (i.e., maximizing SNR in the source solution), eLORETA is a strong choice of algorithm due to zero PAD (Figures 3b and Figure 4a), low levels of leakage (Figures 3c,d and 4b,c), and high robustness to artificial sensor noise (Figure 5). The primary limitation of eLORETA for voxel‐wise analysis is that the source space solution may also have a tendency to explain temporal fluctuations due to background activity (Figure S4b), that is, including non‐neuronal activity in the source estimate. This should be considered whenever using eLORETA for source reconstruction and sensor space data should be appropriately preprocessed to minimize external noise and artifacts as much as possible.

Of the six algorithms studied, there are two that we do not recommend for distributed, whole brain cortical reconstruction of resting‐state MEG based on our results; namely *unweighted* MNE (Hämäläinen & Ilmoniemi, 1994) and *unweighted* (i.e., unit‐gain) LCMV (Van Veen et al., 1997). Although this recommendation is already commonly accepted, here we presented clear evidence specific for resting‐state analyses. While MNE did not perform poorly, it was widely outperformed in almost every measure by wMNE. The resolution metric results match those reported Hauk et al. (2019) and Lin et al. (2006) in this respect. We therefore recommend that wMNE should be preferred to unweighted MNE. Similar reasoning can be applied to recommend that UNGMV should be preferred to LCMV. In particular, LCMV exhibited notably higher PAD/fPAD than other measures (including UNGMV), in line with studies using simulated dipoles (Halder et al., 2019; Pascual‐Marqui et al., 2018). Unweighted LCMV exhibits a depth bias (i.e., peak of activation in the center of the head), which likely explains the large PAD/fPAD. For this reason, Hillebrand, Barnes, Bosboom, Berendse, and Stam (2012) suggested that UNGMV be used for resting‐state data over LCMV due to the depth bias of the latter resulting in a nonuniform projection of sensor noise. However, a potential limitation of the noise‐normalization step in the UNGMV beamformer is a potential bias in multi‐session statistical analysis, and hence for comparing power between conditions LCMV may be more appropriate (Luckhoo, Brookes, & Woolrich, 2014) (or, alternatively, a nonadaptive linear method such as the LSMN methods). Recently, an empirical Bayesian version of the LCMV beamformer (Belardinelli, Ortiz, Barnes, Noppeney, & Preissl, 2012) has been shown to explain approximately 80% of cross‐validated variance in resting‐state data (Little et al., 2018), which is higher than reported in our data (Figure S4), and hence could be a promising choice of beamformer‐based solution.

### 4.2 The reduced HCP‐MMP atlas optimized for MEG

The original HCP‐MMP atlas consists of 360 cortical ROIs delineated by their distinct structural, functional, and connectivity profiles (Glasser et al., 2016), which offers good neuroanatomical precision essential for understanding macroscopic brain network dynamics. As such, the HCP‐MMP atlas has been widely used for parcellation of resting‐state fMRI data (Dermitaş et al., 2019; Dubois et al., 2018; Ito et al., 2017; Preti & Van De Ville, 2019; Watanabe et al., 2019). Nevertheless, the fine spatial resolution of this atlas becomes a key limiting factor for applying it to MEG, because MEG data acquired in a typical scanner with 200–300 sensors would lead to rank deficiency if parcellated into 360 regions.

Here, we presented a reduction of the original HCP‐MMP atlas with 230 cortical ROIs, in which deep regions with lower spatial resolution (chosen using a data‐driven approach based upon the leadfield matrix, see Supporting Information for details) were more coarse grained than in the original atlas. The target of reduction to 230 ROIs was determined because our MEG data consisted of 274 gradiometers, and after preprocessing (including noise projection and artifact rejection through ICA decomposition) had a rank of ≥233, although our method can be extended to reduce the original atlas further. Our approach posited that deeper regions of the brain or those with more radial orientation, which have low SNR (Cho, Vorwek, Wolters, & Knösche, 2015) and low spatial resolution (Liu, Dale, & Belliveau, 2002) for MEG, should contain fewer ROIs than superficial regions. This logic was supported by our spatial analysis of resolution metrics for all source reconstruction algorithms (Figure 4), where voxels with the low resolution (i.e., highest SECT) largely corresponded to those with low leadfield norm, quantified by a significant negative correlation between SECT and leadfield norm (Supporting Information).

**FIGURE 6:**
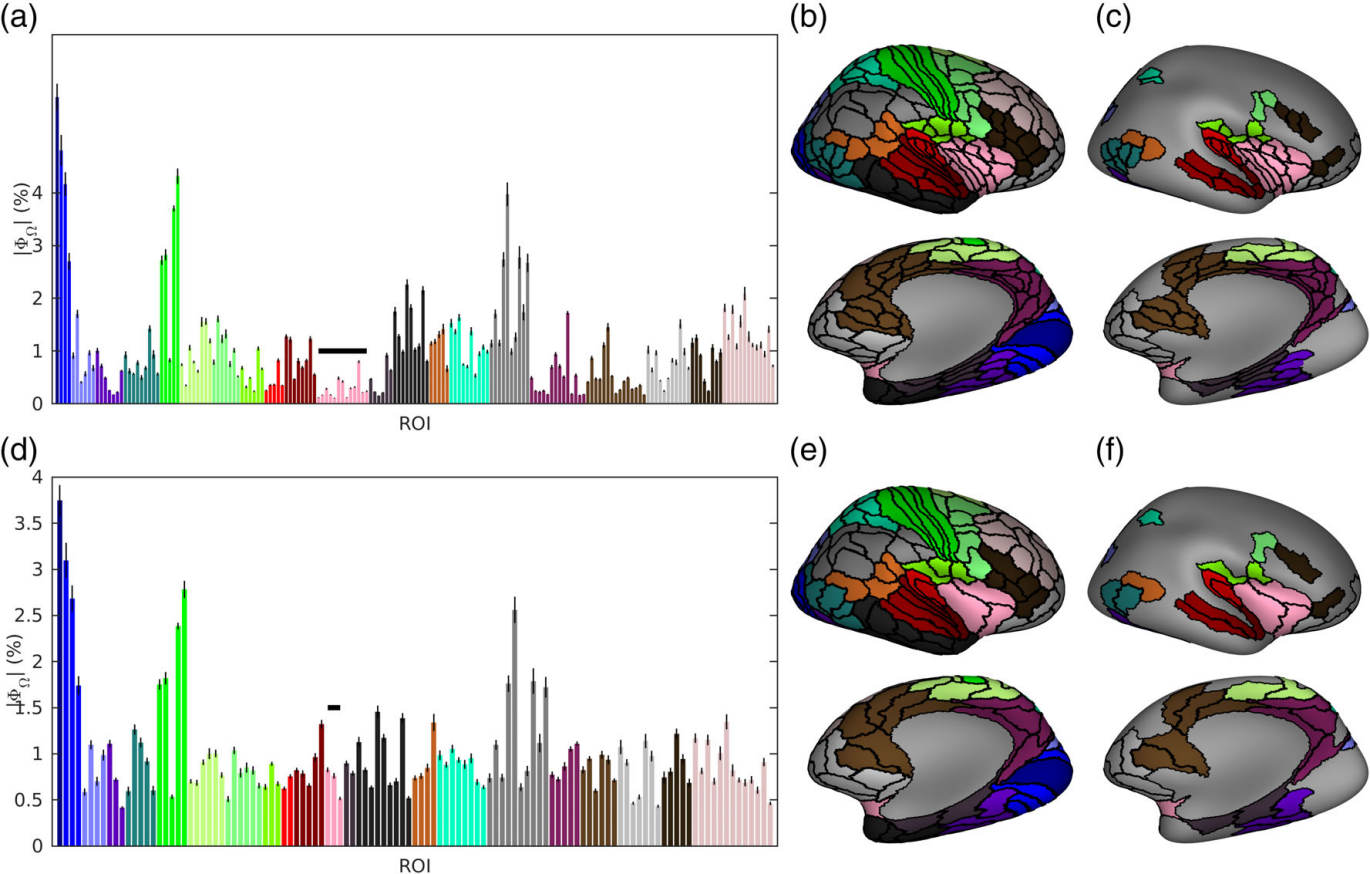
The reduced HCP‐MMP atlas. (a–c) The original atlas (Glasser et al., 2016), consisting of 180 ROIs per hemisphere split into 22 larger “clusters.” (a) The norm of the leadfield, summed over all voxels in an ROI ω (as a percentage of the sum over all ROIs), quantifying the strength with which ω influences the MEG. Clusters are marked by different colors, while each bar is an individual ROI. (b) Each ROI's anatomical location, plotted on the inflated Freesurfer average brain. Top shows the lateral surface, while the bottom is angled to show the medial and ventral surfaces. (c) As in b, but plotting only ROIs, which are modified in the reduced atlas to highlight changes. (d–f) The reduced atlas, consisting of 115 ROIs per hemisphere, plotted similarly as in a–c, respectively. We reduce the atlas by stating that each cluster should have a number of ROIs proportional to its influence on the MEG (a,d). For example, cluster 12 (the insula, the pink cluster marked in a and d by a black bar) has very many weak ROIs, so we combine these ROIs into fewer, larger, more influential ROIs. The result of this is a more uniform distribution of ROI strengths. While the calculation of numbers of ROIs per cluster was automated, the choice of which specific ROIs to be combined was made by hand from studying anatomical and functional closeness, based on the study of Glasser et al. (2016)

We determined the optimal number of ROIs per anatomical cluster for both the 11 participants discussed throughout this manuscript, and validated this result in a larger cohort of 31 participants (including the original 11) with two scans per participant resulting in a total of 62 different leadfield matrices. Figure 6 and Figure S7 show the results for the original and extended cohorts, respectively. While slightly different results may be expected between cohorts due to random sampling, identical numbers of ROIs per cluster were found with both cohorts (Figure S7a). This is likely due to a combination of several factors. First, the key parameter to deciding the number of ROIs was based on the leadfield norms, which were highly consistent between participants (Figure S7c). Furthermore, for each anatomical cluster the leadfield norm statistic was summed over many ROIs (22 clusters covered 360 ROIs), so this procedure likely reduced the influence of small differences between participants. Additionally, small differences between cohorts would be lost during rounding the number of ROIs to integer values.

After determining the optimal number of ROIs per cluster, merging of the regions was done manually within each cluster, based on factors such as whether they were anatomical neighbors (required) and exhibited neuroanatomical and functional similarities (preferred) as reported by Glasser et al. (2016). Particularly, similarities in resting‐state fMRI functional connectivity were prioritized, while properties such as myelination, cortical thickness, and activation during tasks were given low priority in the choice of ROIs to merge. Therefore, while the reduced atlas presented here is appropriate for parcellation of resting‐state activity, one should use it with caution for task evoked MEG.

Alternative approaches exist for parcellating resting‐state MEG data. First, one can opt for functionally derived ROIs, for example, clustering voxels on the basis of functional connectivity profiles (for its application in fMRI data see Yeo et al., 2011). However, this approach is challenging for MEG data, due to the necessity of deciding between a large number of source reconstruction algorithms (Section 2.1), functional connectivity metrics (Dauwels, Vialatte, Musha, & Cichocki, 2010; Wang et al., 2014; Wendling, Ansari‐Asl, Bartolomei, & Senhadji, 2009) and frequency bands (Buzsáki, 2006), each combination of which likely results in a different functional connectivity profile (Hassan et al., 2014, 2017).

Second, one may use atlases for cortical parcellation with various levels of granularity, such as the Destrieux (74 cortical ROIs; Destrieux et al., 2010), AAL (90 cortical ROIs; Tzourio‐Mazoyer et al., 2002), Desikan‐Killiany (168 cortical ROIs; Desikan et al., 2006) and Brainnetome (210 cortical ROIs; Fan et al., 2016) atlases. The reduced HCP‐MMP atlas presented here is advantageous for MEG research in two aspects: (a) it maintains all macroscopic‐level clusters allowing for multi‐modal comparisons with fMRI data; and (b) it maintains the fine grained resolution of the original HCP‐MMP atlas for regions with high SNR in MEG data, while reducing the resolution only in deeper or more radial regions with low SNR in MEG data (Cho et al., 2015; Liu et al., 2002). Therefore, in superficial cortical regions where the MEG estimate is more reliable, the reduced HCP‐MMP atlas has higher resolution than the above coarse‐grained atlases.

Finally, if MEG resting‐state functional connectivity is the aim of the study, an additional alternative is to use the full HCP‐MMP atlas with connectivity metrics insensitive to leakage, such as imaginary part of coherence (Nolte et al., 2004) or (weighted) phase lag index (Stam, Nolte, & Daffertshofer, 2007; Vinck, Oostenveld, van Wingerden, Battaglia, & Pennartz, 2011). For standard connectivity metrics, orthogonalization between pairs of ROIs (Hipp, Hawellek, Corbetta, Siegel, & Engel, 2012) could be used, which does not require full rank data. However, multivariate leakage correction is preferred due to higher reliability of connectivity estimates than pairwise leakage correction (Colclough et al., 2015, 2016), and unlike pairwise correction does not exhibit spurious “ghost” connections (Palva et al., 2018). Therefore, we suggest that the combination of our reduced HCP‐MMP atlas and multivariate leakage correction is a more robust method to estimate resting‐state MEG functional connectomes, as the reduced atlas also takes into account different signal‐to‐noise ratios in MEG data between brain regions. It is worth noting that multivariate leakage correction risks removing zero lag connectivity that is not an artifact of leakage. Recently, Farahibozorg, Henson, and Hauk (2018) presented a split‐and‐merge algorithm to downsample anatomical atlases which alleviates the problem of leakage by merging ROIs based on CTFs derived from the resolution matrix (as opposed to norms of the leadfield presented here). This is potentially an advantage of the adaptive parcellations of Farahibozorg et al. (2018) over the method presented here. Nevertheless, since the CTF depends on the inverse solution, the resulting parcellation from that procedure is likely to also depend on the choice of source reconstruction algorithm. It would be of interest for future work to compare the reduced atlas presented here and an atlas derived following Farahibozorg et al. (2018) using the HCP‐MMP atlas as a start point and source reconstruction algorithms recommended in this study to construct CTFs.

### 4.3 Methodological considerations

A crucial choice that needed to be made for this study was the selection of source reconstruction algorithms to be compared. The list of algorithms studied here is by no means exhaustive, but instead focused on algorithms that may be useful for resting‐state MEG data. Algorithms aimed toward localizing the spatial origin of a small number of active sources (i.e., *source localization*) were excluded, because this is likely to be an unrealistic assumption for resting‐state. Examples of such source localization algorithms include dipole fitting (Scherg, 1990), multiple signal classification (Mosher & Leahy, 1998), and minimum norm estimation using multiple sparse priors (MSP, Friston et al., 2008; Little et al., 2018). Furthermore, we did not include algorithms that may perform well for source reconstructing resting‐state data but are not compatible with our variance explained or resolution analyses, because comparison with the linear inverse algorithms presented here was not possible. These include algorithms which directly estimate resting‐state source space functional networks from the sensor space data without first inverting the data, for example, partial canonical coherence (Popov, Oostenveld, & Schoffelen, 2018; Schoffelen & Gross, 2009), cortical partial coherence (Barzegaran & Knyazeva, 2017), and estimation of MVAR coefficients (Gómez‐Herrero, Atienza, Egiazarian, & Cantero, 2008), as well as linear algorithms estimated in the frequency domain, for example, dynamic imaging of coherent sources (Gross et al., 2001) and frequency domain minimum norm estimates (Yuan, Doud, Gururajan, & He, 2008).

Of the source reconstruction algorithms most commonly used for resting‐state analysis, the majority are covered in the current study. Notable exceptions include dynamic statistical parameter mapping (dSPM; Dale et al., 2000), LORETA (Pascual‐Marqui et al., 1994), and the empirical Bayesian beamformer (EBB; Belardinelli et al., 2012). Comparisons between dSPM and sLORETA have been studied using resolution metrics (Hauk et al., 2011; Hauk et al., 2019; Hedrich et al., 2017) and simulated dipoles (Pascual‐Marqui et al., 2018), generally finding that sLORETA outperforms dSPM in terms of localization error, leakage, and false positive activity. Similarly, in a variance explained analysis of resting‐state MEG, LORETA performed similarly to the MNE solution (Little et al., 2018). The EBB solution is derived from the LCMV beamformer, but is placed in a Bayesian framework in which hyperparameters of the inversion are optimized to increase model fit to the data (Belardinelli et al., 2012; Wipf & Nagarajan, 2009), meaning the variance explained analysis of EBB is not directly comparable with the other algorithms presented here. For these reasons, and to reduce the number of comparisons, dSPM/LORETA/EBB were excluded due to the inclusion of sLORETA/MNE/LCMV (respectively) in this study.

Another crucial methodological decision was choice of methods used to compare different algorithms. Previous studies have compared algorithms for *source localization*—typically evaluating the performance of algorithms for identifying the origin of a small number of sources (Anzolin et al., 2019; Bai et al., 2007; Barzegaran & Knyazeva, 2017; Bonaiuto et al., 2018; Bradley et al., 2016; Finger et al., 2016; Halder et al., 2019; Hassan et al., 2014; Hassan et al., 2017; Hincapié et al., 2017; Pascual‐Marqui et al., 2018; Seeland et al., 2018), such as known networks during task or simulated dipoles. These methods are not directly generalizable to resting‐state data, where activity is not a point source but is distributed widely across the cortex. Instead, for resting‐state data, one can ask to what extent true activity at a certain source location is mislocalized to another point on the cortex. Here, we approached the question of source (mis‐)localization in resting‐state data by considering resolution matrices (Hauk et al., 2011; Hauk et al., 2019; Hedrich et al., 2017). These matrices are the theoretical linear transformation between the true source activity and the estimated activity, and can be used to quantify the performance of a method for all point sources in isolation. For linear inversion methods such as those assessed in this study, the resolution matrix may also be useful to make inferences about distributed point sources. For this reason, resolution metrics are appropriate for analysis of spatial properties of distributed activity such as resting‐state data, and have been suggested for this type of comparison in past literature (Hauk et al., 2011; Hauk et al., 2019; Hedrich et al., 2017).

**FIGURE 7:**
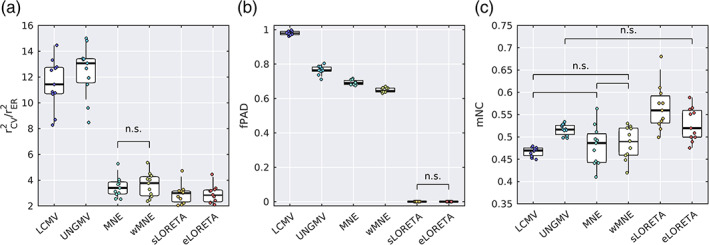
Parcellation based statistics. (a) Ratio of cross‐validated variance explained in empirical data and empty room recordings ($r_{\mathrm{CV}}^{2}/r_{\mathrm{ER}}^{2}$) for parcellated data. (b) Fractional peak activity displacement (fPAD). (c) Mean neighbor correlation (mNC) of ROI time series. For all figures, group‐wise analyses demonstrated a significant effect of algorithms. In these figures, pairwise analyses identified significant differences for all pairs except those marked as nonsignificant (n.s.), following false discovery rate correction. The meaning of whiskers, boxes, and so on are explained in Figure 3

Since the resolution matrices depend on the inverse model, which in itself is data dependent (e.g., data covariance and SNR for the beamformers, noise covariance and SNR for the LSMN methods), we highlight here that results for different data sets (e.g., evoked activity, different cognitive states, clinical cohorts, etc.) may not be equivalent to those presented here. Notably, Hauk et al. (2019) excluded the LCMV beamformer from their resolution analysis because of the dependence on data covariance. Here we chose to include beamformers under the proviso that these results are applicable to resting‐state MEG and may not generalize to other datasets. This was motivated by the fact that in our resting‐state data from healthy adults, there was a correlation between edges of the data covariance matrices of 0.8121±0.0329 (mean ± standard error across pairs of participants), suggesting high consistency across participants. Therefore, the rank ordering of algorithms in terms of a given resolution metric presented here is likely to be consistent in resting‐state MEG datasets recorded from healthy participants, but care should be taken in relating these results to task‐based datasets with different statistical properties. Furthermore, we additionally highlight that the quantitative results presented in Figures 3b–d and 7b are based upon averages across all dipoles, while distributions of resolution metrics are heterogeneous across the cortex (Figure 4). Therefore, for studies focused on a particular source/region of interest as opposed to whole‐brain analyses, one should consider the resolution metrics purely about that source as shown in Figure 4.

There exists a large number of resolution metrics (Hauk et al., 2019). The current study focused on PAD, SEPS, and SECT to address crucial questions of interest. First, given true activity in the cortex, how accurate is the placement of this activity in the source estimate? This question can be addressed quantitatively by “localization accuracy” metrics applied (Hauk et al., 2019), for which dipole PAD was measured in this study (called localization error in previous studies (Hauk et al., 2011; Hauk et al., 2019; Hedrich et al., 2017)). Care must be taken when interpreting the PAD in the resting‐state paradigm, motivating the use of the terminology PAD instead of the localization error terminology commonly used in source localization studies. We are not attempting to quantify the difference in peak activation of the estimate given a single “true” active dipole as in simulation or theoretical studies (Barzegaran & Knyazeva, 2017; Halder et al., 2019; Pascual‐Marqui, 2007; Pascual‐Marqui et al., 2018), because of the expected cross talk from distributed sources in resting‐state data. As a result, “true” activity at a certain location does not necessarily imply a peak of estimated activity at that location—for example, localization statistics based on peak activation for sLORETA are accurate for single dipole localization, but are imperfect for localizing multiple dipoles (Bradley et al., 2016). The PAD statistic used in this study is therefore more properly interpreted as follows: given true activity at a dipole, that activity will most strongly influence dipoles approximately PAD cm from the true source on average, regardless of activity at other locations.

The second key question we chose to address with resolution metrics was related to source leakage. More properly, we addressed two questions related to leakage. First, given an estimate of activity at a given dipole, how strongly is this estimate influenced by leakage from other dipoles? Second, given that there is activity at a given dipole, how strongly does this leakage from this dipole influence the estimate at other dipoles? These questions are important for resting‐state functional connectivity analysis (Colclough et al., 2016), where spurious functional connections may arise due to leakage (Colclough et al., 2015). They can be addressed by “spatial extent” metrics applied to the CTF and PSF, respectively (Hauk et al., 2019). Here, we chose a metric which has been called “spatial dispersion” in past studies (Hauk et al., 2011; Hauk et al., 2019; Hedrich et al., 2017). When applied to the PSF this metric describes how activity at a given location “disperses” due to leakage (hence, the name SD), but when applied to the CTF this metric describes the spatial extent to which other locations influence the seed vertex through leakage. Hence, the name “spatial extent of cross talk” was used here to avoid confusion in interpretation. For consistency, we use the name “spatial extent of point spread” for SD applied to the PSF.

The aim of the resolution analysis is to compare the estimated source activity with the true source activity, based on the linear transformation that theoretically relates them. A complementary method of comparing the solutions, highlighting temporal as well as spatial properties, would be to test the temporal variance of source data explained by the estimate. This is usually not possible for empirical MEG data since the true source dynamics is unknown in the absence of simultaneous intracranial recordings. However, because the forward mapping has a unique solution for given source dynamics, (cross‐validated) temporal variance explained $r_{\mathrm{CV}}^{2}$ between the forward mapped source solution and the measured MEG data can be viewed as a proxy for accuracy of the source space solution. Indeed, Bonaiuto et al. (2018) found high correlations (.98–1) between cross‐validated sensor space errors and source space free energy for simulated dipoles. Sensor space temporal variance explained is particularly useful for quantifying the quality of source reconstruction of resting‐state data, since it makes no assumptions on the number or locations of active sources (i.e., can be applied to distributed cortical activity), is a whole brain measure as opposed to studying an individual ROI (Bonaiuto et al., 2018), and considers not only spatial localization, but also temporal accuracy of the reconstructed time courses.

However, there are also limitations to sensor space variance explained as a metric in empirical resting‐state data. Resting‐state sensor MEG data is typically low SNR, containing external noise and non‐neuronal artifacts. An ideal source reconstruction should not explain this non‐neuronal data, and hence should not fully explain the variance of the data. Without an accurate measure of the SNR of the data, it is therefore impossible to find the “correct” amount of variance explained (above which any variance explained is overfitting of noise). To address these issues, we used the measure $r_{\mathrm{CV}}^{2}/r_{\mathrm{ER}}^{2}$ in this study, which is the ratio of cross‐validated temporal variance explained in the resting‐state data against the same measure in empty‐room data. Since the latter contains no neuronal data, it gives a measure of the degree to which an algorithm overfits to nonbrain sources, and by normalizing against this value we correct for non‐neuronal artifacts and noise. To further clarify this normalization procedure, consider the case of an unregularized MNE which—ignoring cross‐validation for the purposes of this example—should explain 100% of the variance of any data, giving $r^{2}=1$. Without normalization by empty room data, and treating raw temporal variance explained as a measure of quality of the solution, the unregularized MNE could be viewed as a “perfect” solution. However, given empty‐room data, the unregularized MNE will also explain 100% of the variance, demonstrating that the solution attributes non‐neuronal data to brain activity and hence the estimated activity cannot be trusted to be primarily real source dynamics. Conversely, the beamformers explained much less of the resting‐state data (approximately 55–75%), but explained essentially none of the empty room data (4–8%), suggesting the estimated temporal fluctuations in the resting‐state data is likely to be largely “real” source dynamics. Without the normalization, unregularized MNE ($r^{2}=1$) would perform “better” than the beamformers ($r^{2}=0.55-0.75$), while the normalization accounts for this overfitting, with unregularized MNE having $r^{2}/r_{\mathrm{ER}}^{2}=1$ and the beamformers having $r^{2}/r_{\mathrm{ER}}^{2}=11.5-12.3$. Cross‐validation will somewhat aid with this issue, but even with this procedure the LSMN methods explained approximately 20–40% of the empty‐room data, highlighting the importance of the normalization step.

In this study, 30 s epochs were used. Our results should not have a strong dependence on epoch length due to the instantaneous and linear nature of the forward model/inverse solutions. However, sample size bias in the estimation of data covariance may effect beamformer inverses. Following the rule of thumb suggested by Van Veen et al. (1997), for our data a minimum of 800–1,000 samples (3.1–3.9 s) should be used, and hence 30 s is a suitably large epoch length. Similarly, estimation of temporal variance explained statistics ($r_{\mathrm{CV}}^{2}$ and $r_{\mathrm{ER}}^{2}$ may have a sample size bias. Since all algorithms used the same epoch, any sample size bias is equivalent for all algorithms and hence should not influence our results.

Finally, some settings in the source reconstruction pipeline may affect our results. Examples include the method used (including level of detail) for constructing the forward model (Hallez et al., 2007), the use of template models instead of subject specific models (Fuchs, Kastner, Wagner, Hawes, & Ebersole, 2002; Henson, Mattout, Phillips, & Friston, 2009), and the density of the source space (Henson et al., 2009). In particular, the resolution metrics are likely to be dependent on these factors, due to the dependence of the resolution matrix on the leadfield matrix. Errors in the forward model are additionally known to affect the cross‐validated variance explained (Little et al., 2018). By extension, since MEG and EEG give complementary information (Ding & Yuan, 2013) and the forward model is constructed differently between these modalities (Mosher, Leahy, & Lewis, 1999), it should not be taken for granted that the results presented here apply to EEG resting‐state data. Hence, the generalizability of our results to different acquisition modalities and robustness to differences in the forward model is an open question. Importantly, in this study, the methodology was consistent across participants, and all statistics were performed within participants. Therefore, our comparisons between algorithms are unlikely to be biased or influenced by such confounding factors.

### 4.4 Conclusions

In conclusion, there is no “one size fits all” algorithm for source reconstructing resting‐state data. Table 1 outlines the strengths and weaknesses of each algorithm studied here, and makes recommendations on the appropriate situations in which each algorithm should be applied. When parcellating distributed source data for ROI‐level analysis, we recommend eLORETA (Pascual‐Marqui, 2007, 2009) in combination with our new MEG‐optimized reduction of the high‐resolution HCP‐MMP atlas (Glasser et al., 2016) as an appropriate methodology for resting‐state MEG. These conclusions are supported by extensive quantitative evaluation using a range of metrics including measures of variance explained and resolution properties of the inverse.

## Supporting information

**AppendixS1.** Supporting Information.

## Data Availability

Data preprocessing and source reconstruction used Fieldtrip version 2019‐07‐16 (\url{http://www.ru.nl/neuroimaging/fieldtrip}). Processed data were analysed using a set of custom scripts written in MATLAB R2017a, which are open‐source and freely available in a GitHub repository (\url{https://github.com/lukewtait/evaluate_inverse_methods}). Our reduced HCP atlas is freely available for download in the same Github repository. Data used in this study are available upon request.

## APPENDIX A MATHEMATICAL FORMULATION OF SOURCE RECONSTRUCTION ALGORITHMS

In this appendix, we provide the mathematical framework for the source reconstruction algorithms used in the manuscript. All algorithms involve estimating the inverse matrix Φ (whose rows are called spatial filters) in Equation 2, which is an inverse of the leadfield matrix L in the forward model (Equation 1).

### Beamformers

Beamformers calculate the filter for each dipole individually. Let us denote the column of the leadfield matrix corresponding to a dipole i as $L_{i}\in {\mathbb{R}}^{N_{x}\times 1}$. Then we define the beamformer weights for this dipole as $\Phi_{i}\in {\mathbb{R}}^{1\times N_{x}}$, which can be used to construct an inverse matrix Φ with spatial filters $\Phi_{i}$.

#### LCMV beamformer

The linearly constrained minimum‐variance (LCMV) beamformer (Van Veen et al., 1997) makes two constraints. First, to ensure that Φ is the inverse of L and has unit gain, we require $\Phi_{i}L_{i}=1$. Second, to ensure zero response at other locations in the source space, we wish to attain $\Phi_{i}L_{j}=0$ for i≠j. In practice this second constraint cannot be met exactly, but instead an optimal spatial filter can be designed which minimizes the variance of the output of the filter while still satisfying the first constraint. Mathematically, this problem is to minimize $\mathrm{tr}\left(\Phi_{i}C\Phi_{i}^{T}\right)$, where C is the data covariance, subject to $\Phi_{i}L_{i}=1$, and has the solution

(A1) $$\Phi_{\text{LCMV},i}={\left(L_{i}^{T}{\left[C+\mathrm{\lambda I}\right]}^{-1}L_{i}\right)}^{-1}L_{i}^{T}{\left[C+\mathrm{\lambda I}\right]}^{-1}.$$

Here, λ is a regularization parameter used to invert C to avoid issues arising due to potential rank deficiency.

#### UNGMV beamformer

The LCMV filter is known to mislocalize superficial sources to deep locations due to nonuniform projections of sensor noise (Hillebrand et al., 2012). To account for this, the beamformer weights can be normalized to unit vector norm (Hillebrand et al., 2012). Here, we call this solution the *unit‐noise‐gain minimum variance beamformer* (UNGMV,Sekihara & Nagarajan, 2015), given by

(A2) $$\Phi_{\text{UNGMV},i}=\frac{\Phi_{\text{LCMV},i}}{∥\Phi_{\text{LCMV},i}∥}.$$

### LSMN estimates

While beamformers consider each source point individually, regularized LSMN type solutions reconstruct all sources within the source space simultaneously. An unregularized LSMN estimate aims to minimize $∥x-L\hat{s}{∥}^{2}$, which has the Moore‐Penrose psuedo‐inverse $\Phi =L^{T}\left({\mathrm{LL}}^{T}\right)$ as a solution. However, since the problem is typically ill‐posed, with $N_{s}\gg N_{x}$, often this matrix Φ is singular. Regularization is often used to alleviate this issue, instead minimizing $∥x-L\hat{s}{∥}^{2}+\lambda x^{T}\mathrm{Wx}$. Here, λ is a regularization parameter which controls the extent to which the solution is regularized, and W is a weight matrix corresponding to a prior estimate of source variance–covariance (Dale et al., 2000; Hassan et al., 2014). The solution to this minimization is

(A3) $$\Phi ={\mathrm{WL}}^{T}{\left({\mathrm{LWL}}^{T}+\lambda I\right)}^{-1}.$$

The MNE, wMNE, and eLORETA solutions all take this form, differing only in the prior estimate of source covariance W.

#### MNE

The solution often known simply as the minimum norm estimate (MNE; Hämäläinen & Ilmoniemi, 1994) makes a uniform estimate of source covariance, and is given by

(A4) $$\Phi_{\mathrm{MNE}}=\Phi ,\;W=I.$$

#### wMNE

The MNE solution suffers from depth bias, so the *weighted minimum norm estimate* sets the diagonals of W inversely proportional to the norm of the leadfield (Fuchs et al., 1999; Lin et al., 2006), that is,

(A5) $$\Phi_{\text{wMNE}}=\Phi ,\;W_{\mathrm{ij}}=\left(\begin{array}{cc} {\left(L_{i}^{T}L_{i}\right)}^{-1/2} & \text{if}\;i=j\\ 0 & \text{if}\;i\neq j \end{array}\right..$$

This solution essentially assumes as a prior that sources, which only weakly influence the M/EEG must have a higher variance to be picked up by the sensors.

#### eLORETA

The *exact low resolution electromagnetic tomography* (eLORETA) solution (Pascual‐Marqui, 2007, 2009) optimizes the weights such that not only is depth bias accounted for, the solution attains theoretically exact localization (Pascual‐Marqui, 2007). The eLORETA solution is given by

(A6) $$\Phi_{\text{eLORETA}}=\Phi ,\;W_{\mathrm{ij}}=\left(\begin{array}{cc} {\left(L_{i}^{T}{\left({\mathrm{LW}}^{-1}L^{T}+\lambda I\right)}^{+}L_{i}\right)}^{-1/2} & \text{if}\;i=j\\ 0 & \text{if}\;i\neq j \end{array}\right..$$

An iterative algorithm, described by Pascual‐Marqui (2007), can be used to find these weights numerically.

#### sLORETA

Finally, an alternative approach to account for depth bias in the source localization is to estimate standardized distributions of current density, normalized by expected variance of each source. *Standardized low resolution electromagnetic tomography* (sLORETA; Pascual‐Marqui, 2002) first calculates $\hat{s}=\Phi_{\mathrm{MNE}}x$ and then normalizes each time point to standardized variance. The sLORETA solution is therefore

(A7) $$\Phi_{\text{sLORETA}}={\left(\mathrm{diag}\left(\Phi_{\mathrm{MNE}}L\right)\right)}^{-1/2}\Phi_{\mathrm{MNE}}.$$

While this standardization reduces the depth bias, the resulting solution can no longer be interpreted as a measure of the intensity of current density, and is more appropriately interpreted as probability of source activation.

### Regularization and the signal‐to‐noise ratio

For fair comparison between algorithms, λ must be chosen consistently for all algorithms (Hauk et al., 2019). One such way to do so is based on the SNR of the data (Hauk et al., 2019; Lin et al., 2008). For beamformers, the relationship between SNR and λ is

(A8) $$\lambda =\frac{\text{trace}\left(C\right)}{N_{x}\cdot {\mathrm{SNR}}^{2}}.$$

For LSMN type solutions, the relationship is

(A9) $$\lambda =\frac{\text{trace}\left({\mathrm{LWL}}^{T}\right)}{N_{x}\cdot {\mathrm{SNR}}^{2}}.$$

Note that the $N_{x}$ term in both denominators is derived from the trace of the noise covariance, which throughout this manuscript (and in all equations for inverse solutions above) is a priori estimated to be λI. Given an arbitrary noise covariance matrix λ∑, the denominators should be replaced with $\text{trace}\left(\sum \right)\cdot {\mathrm{SNR}}^{2}$ (Lin et al., 2008).

## APPENDIX B THE REDUCED HCP‐MMP ATLAS

### Algorithm to identify the optimum number of ROIs per cluster

Below, we show a MATLAB code demonstrating the algorithm to identify the optimum number of ROIs per cluster. The strength of each of the 22 clusters Ω (i.e., $∥L_{\Omega }∥$ in Equation 10 and normLFW in the code below; a 22×1 vector), the target number of regions (targetN in the code below; a scalar, here 125), and the number of ROIs per cluster in the original atlas (n_orig; a 22×1 vector) should be taken as input.

[function n_new = determine_nROIs_per_cluster(normLFW,n_orig,targetN)

% Normalize the strengths of the regions to unit sum

normLFW = normLFW/sum(normLFW) ;

% Ideally, the number of ROIs per region is proportional to the strength

n_new = round(targetN*normLFW) ;

% We don’t wish to split regions, only merge. Therefore we don’t want any

% regions to have a larger value in n_new than in n_orig

i_more = find(n_new > n_orig) ; % List of regions with larger value in

% n_new than in n_orig

while ~isempty(i_more) % While there are regions with too many ROIs

% Reset those regions to have the original number of ROIs

n_new(i_more) = n_orig(i_more) ;

% Recalculate target number of ROIs, ignoring those we are fixing

% to the original value

targetN = targetN ‐ sum(n_orig(i_more)) ;

% Re‐normalize strengths of ROIs to unity, ignoring those we are

% fixing to the original value

normLFW(i_more) = nan ;

normLFW = normpLFW/nansum(normLFW) ;

% Recalculate number of ROIs in regions we are not setting to the

% original value

n_new = round(targetN*normLFW) ;

n_new(i_more) = n_orig ;

% Now we have a new n_new, once again calculate list of regions

% with larger n_new than n_orig

i_more = find(n_new./n_orig > 1) ;

end

### Reducing the atlas

The algorithm above gives a target number of ROIs per cluster. To attain this target, we then examined each cluster and manually merged ROIs until the target number was achieved. To do so, the following criteria were used (in order of priority):

1. Merged ROIs *must* be in the same cluster.

2. Merged ROIs *must* be anatomical neighbors.

3. The resulting strengths of the merged ROIs should be approximately uniform. For example, consider a cluster with three ROIs, each of which neighbor both other ROIs (i.e., criteria 1 and 2 are satisfied). The ROIs have strengths 0.8, 0.4, and 0.3, and the target number of ROIs is two. We prefer to merge the ROIs with strength 0.4 and 0.3 (resulting in ROIs with strengths 0.8 and 0.7) than, for example, the ROIs with strength 0.8 and 0.4 (resulting in ROIs with strengths 1.2 and 0.3). This result is justified, since we found from our resolution analysis that all algorithms demonstrated significant negative correlations between leakage (SECT) and the leadfield norm (LCMV/UNGMV/sLORETA r=−.29; MNE r=−.66, wMNE r=−.69, and eLORETA r=−.68), suggesting that weaker sources have lower resolution, and therefore weaker ROIs should be merged to form larger but stronger ROIs.

4. Merged ROIs should display neuroanatomical and functional similarities, particularly having a low gradient in resting‐state functional connectivity on the boundaries between the ROIs. These decisions were based on the detailed neuroanatomical descriptions given in supporting information 3 of Glasser et al. (2016), in which a wide range of structural and functional imaging modalities were described for each cluster.

Supporting Information contains a list of all regions in the reduced atlas, and the choice of which ROIs were merged.
