# Supplementary material for: A systematic evaluation of source reconstruction of resting MEG of the human brain with a new high‐resolution atlas: Performance, precision, and parcellation
Source: Hum Brain Mapp. 2021 Jul 5;42(14):4685–707. doi: 10.1002/hbm.25578 (PMC8410546; doi:10.1002/hbm.25578)
Supplement: Supplementary file 1 — AppendixS1. Supporting Information. [file HBM-42-4685-s001.pdf]

Supplementary Material for Tait et al. *"Towards optimal source reconstruction of resting MEG of the human brain: performance, precision, and parcellation"*

Supplementary Tables

	mean	sem	Post-hoc pairwise comparisons (p-values)				
			UNGMV	MNE	wMNE	sLORETA	eLORETA
LCMV	11.480	0.536	0.0015	0.0015	0.0015	0.0015	0.0015
UNGMV	12.354	0.571		0.0015	0.0015	0.0015	0.0015
MNE	2.786	0.177			0.0015	0.0620	0.1318
wMNE	2.772	0.173				0.0085	0.8311
sLORETA	2.840	0.189					0.0027
eLORETA	2.771	0.176					

Supplementary Table S1: Means and standard errors on the mean (sem) of r^2_{CV}/r^2_{ER}, and Benjamini-Hochberg corrected p-values for post-hoc pairwise comparisons between algorithms (see Figure 3A). In the text, significant differences correspond to corrected $p < 0.05$.

	mean (cm)	sem (cm)	Post-hoc pairwise comparisons (p-values)				
			UNGMV	MNE	wMNE	sLORETA	eLORETA
LCMV	5.20	0.14	0.0012	0.0012	0.0012	0.0012	0.0012
UNGMV	1.57	0.04		0.0450	0.0023	0.0012	0.0012
MNE	1.12	0.01			0.0012	0.0012	0.0012
wMNE	1.41	0.01				0.0012	0.0012
sLORETA	0	0					1
eLORETA	0	0					

Supplementary Table S2: Means and standard errors on the mean (sem) of PAD, and Benjamini-Hochberg corrected p-values for post-hoc pairwise comparisons between algorithms (see Figure 3B). In the text, significant differences correspond to corrected $p < 0.05$.

	mean (cm)	sem (cm)	Post-hoc pairwise comparisons (p-values) UNGMV	MNE	wMNE	sLORETA	eLORETA
LCMV	4.60	0.04	1	0.0010	0.0010	0.0010	0.0010
UNGMV	4.60	0.04		0.0010	0.0010	0.0010	0.0010
MNE	3.70	0.03			0.0010	0.0010	0.0010
wMNE	3.60	0.02				0.0010	0.0010
sLORETA	3.69	0.03					0.0010
eLORETA	3.79	0.03					

Supplementary Table S3: Means and standard errors on the mean (sem) of SECT, and Benjamini-Hochberg corrected p-values for post-hoc pairwise comparisons between algorithms (see Figure 3C). In the text, significant differences correspond to corrected $p < 0.05$.

	mean (cm)	sem (cm)	Post-hoc pairwise comparisons (p-values) UNGMV	MNE	wMNE	sLORETA	eLORETA
LCMV	5.29	0.08	0.0010	0.0010	0.0010	0.0010	0.0010
UNGMV	4.65	0.05		0.0010	0.0010	0.0010	0.0010
MNE	3.70	0.03			0.0068	0.0010	0.0010
wMNE	3.69	0.03				0.0010	0.0010
sLORETA	4.23	0.03					0.0010
eLORETA	3.69	0.03					

Supplementary Table S4: Means and standard errors on the mean (sem) of SEPS, and Benjamini-Hochberg corrected p-values for post-hoc pairwise comparisons between algorithms (see Figure 3D). In the text, significant differences correspond to corrected $p < 0.05$.

	mean	sem	Post-hoc pairwise comparisons (p-values) UNGMV	MNE	wMNE	sLORETA	eLORETA
LCMV	11.492	0.573	0.0146	0.0011	0.0011	0.0011	0.0011
UNGMV	12.469	0.606		0.0011	0.0011	0.0011	0.0011
MNE	3.462	0.239			0.0674	0.0011	0.0011
wMNE	3.626	0.285				0.0011	0.0011
sLORETA	2.911	0.227					0.0011
eLORETA	2.915	0.205					

Supplementary Table S5: Means and standard errors on the mean (sem) of r^2_{CV}/r^2_{ER} in the parcellated data, and Benjamini-Hochberg corrected p-values for post-hoc pairwise comparisons between algorithms (see Figure 7A). In the text, significant differences correspond to corrected $p < 0.05$.

	mean	sem	Post-hoc pairwise comparisons (p-values) UNGMV	MNE	wMNE	sLORETA	eLORETA
LCMV	0.980	0.003	0.0010	0.0010	0.0010	0.0010	0.0010
UNGMV	0.765	0.008		0.0010	0.0010	0.0010	0.0010
MNE	0.692	0.004			0.0010	0.0010	0.0010
wMNE	0.649	0.004				0.0010	0.0010
sLORETA	0	0					1
eLORETA	0	0					

Supplementary Table S6: Means and standard errors on the mean (sem) of fPAD in the parcellated data, and Benjamini-Hochberg corrected p-values for post-hoc pairwise comparisons between algorithms (see Figure 7C). In the text, significant differences correspond to corrected $p < 0.05$.

	mean	sem	Post-hoc pairwise comparisons (p-values) UNGMV	MNE	wMNE	sLORETA	eLORETA
LCMV	0.467	0.003	0.0016	0.3652	0.2772	0.0016	0.0016
UNGMV	0.516	0.004		0.0186	0.0186	0.0016	0.2982
MNE	0.481	0.013			0.1270	0.0016	0.0016
wMNE	0.487	0.011				0.0016	0.0016
sLORETA	0.566	0.015					0.0016
eLORETA	0.528	0.011					

Supplementary Table S7: Means and standard errors on the mean (sem) of mNC in the parcellated data, and Benjamini-Hochberg corrected p-values for pairwise comparisons between algorithms (see Figure 7D). In the text, significant differences correspond to corrected $p < 0.05$.

Supplementary Text S1. List of regions in the reduced HCP-MMP atlas

In the sections below, we describe which ROIs were merged and how this decision was made for each cluster. The order of the clusters described here matches those of Figure 6A and D from left to right, i.e. S1.4 Ventral stream visual cortex corresponds to purple in Figure 6A and D, since this is the fourth colour to appear from left to right. All neuroanatomical and functional descriptions used to guide our reduction, such as resting-state functional connectivity, myelination, cortical thickness, and activation during tasks, are taken from Supplementary Material 3 of Glasser et al. (2016). ROI strengths reported here are based on the analysis of 62 scans from 31 participants. Supplementary Figure S7 demonstrates these results are consistent with results derived from 11 participants and shown in Figure 6.

S1.1. Primary visual cortex (V1)

In the original atlas, this first cluster contains only a single ROI, the primary visual cortex (V1). Regardless of the fact it only contains a single ROI, V1 is the strongest cluster. In the initial iteration of the algorithm, V1 was actually ideally predicted to contain four ROIs. However, since we are aiming to reduce the number of ROIs, we do not split ROIs. Therefore V1 was not altered in the reduced atlas.

S1.2. Early visual cortex

The early visual cortex contains the second, third, and fourth visual areas (V2, V3, V4). Like cluster 1, cluster 2 had very high strength, and in the initial iteration of the algorithm was predicted to have an optimum number of 7 ROIs. Accounting for the fact we do not split ROIs, the final iterate of the algorithm suggested three ROIs was optimum, and hence cluster 2 was unaltered in the reduced atlas.

S1.3. Dorsal stream visual cortex

In the original atlas, the dorsal visual stream contains six ROIs, including five visual areas (V3A, V3B, V6, V6A, V7) and an intraparietal sulcus area (IPS1). We found the strengths of each ROI to be in the range 0.2107% to 0.9239%. Our algorithm identified the optimum number of ROIs to be four. Across the dorsal stream visual cortex, there was relative homogeneity in resting-state functional connectivity, with primary differences between ROIs being in terms of myelination and activation during a battery of tasks. We therefore merged V3B (0.2107%) with its neighbour V7 (0.3667%; total strength 0.5774%), which have no resting-state FC gradient and differ from neighbouring areas V6A, IPS1, IP0 and V3CD in having more myelin. We additionally merged V6 (0.5571%) and V6A (0.3201%; total strength 0.8772%), which differed only in working memory and MOTOR AVG tasks. The resulting range of strengths was 0.4926% to 0.9239%. The resulting four regions were therefore V3A, V3B+V7, V6+V6A, IPS1.

S1.4. Ventral stream visual cortex

In the HCP-MMP atlas, the ventral visual stream contains seven ROIs including visual area 8 (V8), the ventral visual complex (VVC), the PIT complex (PIT), the fusiform face complex (FFC), and the ventromedial visual areas 1-3 (VMV1, VMV2, and VMV3). We found the strengths of each ROI to be in the range 0.0992% to 0.6106%. Our algorithm identified the optimum number of ROIs to be three. Across the ventral stream visual cortex, there was relative homogeneity in resting-state functional connectivity, with primary differences between ROIs being in terms of functional activation during a battery of tasks. V8 (0.2883%) and VVC (0.3719%) were reported by Glasser et al. (2016) to be the heavily myelinated core of the ventral visual stream, differing only by a visuotopic boundary and cortical thickness, so we chose these two ROIs to merge (total strength 0.6602%). Glasser et al. (2016) additionally reported the only significant difference between PIT (0.3995%) and FFC (0.6105%) was for cortical thickness, so these two ROIs were merged (total strength 1.0101%). Finally, we merged the neighbouring ventro-medial visual areas VMV1 (0.1489%), VMV2 (0.0992%), and VMV3 (0.1193%; total strength (0.4474%). The resulting range of strengths was 0.4474% to 1.0101%. Whilst this is quite a broad range, we were limited by the anatomical organization of this cluster; VMV1-3 form a strip, with the only anatomical neighbours being VVC and V8 on the border of V3, whilst FFC and PIT lay on the opposite side of the VVC+V8 complex.

This means achieving homogeneity in the strengths of the merged regions whilst achieving neuroanatomical and functional similarity was not possible. The resulting three regions were therefore V8+VVC, VMV1-3, PIT+FFC.

S1.5. MT+ complex and neighbouring visual areas

Cluster 5 contains nine ROIs including four in the MT+ complex (MT, MST, V4t, and FST), the adjacent area PH, three lateral occipital areas (LO1, LO2, and LO3), and their adjacent area V3CD. We found the strengths of each ROI to be in the range 0.2578% to 0.8503%. Our algorithm identified the optimum number of ROIs to be five. Homoeogeneity of the MT+ complex in terms of resting-state functional connectivity has been reported; particularly the MT/MST boundary was reported to be less robust than most others reported by Glasser et al. (2016), differing primarily in terms of a single RANDOM-TOM task. The MST/MT regions additionally differed from the rest of the MT+ complex primary due to activation in the BODY-AVG task, we no difference in resting-state functional connectivity. We therefore merge MT (0.3798%), MST (0.2578%), and V4t (0.2986%; total strength (0.9362%). We also merged the three lateral occipital areas LO1 (0.3597%), LO2 (0.3324%), and LO3 (0.4191%; total strength 1.1112%), which differed in cortical thickness and a battery of tasks. The resulting range of strengths was (0.5053% to 1.1112%), and the resulting five regions were MT+MST+V4t, FST, PH, LO1-3, V3CD.

S1.6. Somatosensory and motor cortex

The somatosensory and motor cortex contains the cortical Brodmann areas 1, 2, 3a, 3b, and 4. The initial iterate of the algorithm predicted cluster 6 should contain 10 ROIs, so like clusters 1, 2, and 17, this cluster is actually over represented in the reduced parcellation. Accounting for the fact we do not split ROIs, the final iterate of the algorithm suggested five ROIs was optimum, and hence cluster 6 was unaltered in the reduced atlas.

S1.7. Paracentral lobular and mid-cingulate cortex

Cluster 7 contains eight ROIs including the dorsal and ventral cingulate motor areas 24d (24dd and 24dv), supplementary motor areas (6mp, 6ma, SCEF), and subdivisions of area 5 (5m, 5L, 5mv). The strengths of these ROIs were in the range 0.1772% to 0.8865%. Our algorithm identified the optimum number of ROIs to be 6. Here, we considered the distributions of strengths of ROIs. The supplementary motor areas had strengths in the range 0.6430-0.8865%, whilst area 5 had strengths in the range 0.3427-0.5480, and area 24d had strengths 0.1772% (24dv) and 0.3911% (24dd) respectively. Therefore, whilst Glasser et al. (2016) reported a small difference in resting-state functional connectivity between 24dd and 24dv, this was also notably the weakest set of regions and hence in the interest of uniformity in ROI strengths we merged these two regions (total strength 0.5683%). Similarly, we merged areas 5m (0.4241%) and 5mv (0.3472%; total strength 0.7713%). The resulting range of strengths was 0.5480% to 0.8865%. The seven regions in the resulting merged atlas were therefore 24dd+24dv, 6mp, 6ma, SCEF, 5m+5mv, and 5L.

S1.8. Premotor cortex

The premotor cortex contains seven ROIs including area 55b (55b), the superior premotor regions (6d, 6a) and frontal eye field (FEF), and the inferior premotor regions (6v, 6r) and premotor eye field (PEF). The strengths of these regions ranged from 0.2908% to 0.8795%. Our algorithm suggested this cluster should contain six ROIs. PEF (0.2908%) had notably lower strength than the other ROIs in this cluster, so was a natural choice to merge. The next weakest ROIs were 6v (0.4566%) and 55b (0.4591%), which were both anatomical neighbours of PEF and therefore strong candidates for ROIs to merge. 55b exhibited a notably stronger gradient in resting-state functional connectivity along the border of PEF than 6v did (Glasser et al., 2016), hence we merged PEF and 6v (total strength 0.7474%). The resulting range of strengths 0.4591% to 0.8795%, and the resulting ROIs were 55b, 6d, 6a, FEF, PEF+6v, and 6r.

S1.9. Posterior opercular cortex

The posterior opercular cortex contains six ROIs. These are area 43 (43), the frontal opercular area 1 (FOP1), opercular areas 1-4 (OP1, OP2-3, OP4), and area PFcm (PFcm). These regions range in strength from 0.1362% to 0.6347%. The algorithm suggested the optimum number of ROIs was three. FOP1 (0.1846%) was one of the weakest areas and had a single anatomical neighbour 43 (0.4058%) with a small difference in functional connectivity and a large difference in activation during the motor task (Glasser et al., 2016), so we chose to merge these ROIs (total strength 0.5904%). We additionally merged areas OP1 (0.2690%), OP2-3 (0.1362%) and PFcm (0.3614%; total strength 0.7666%), Between these areas, the primary differences are in activation during tasks such as language and motor, cortical thickness, and a small gradient in functional connectivity. The resulting range of strengths was 0.5904% to 0.7666%. The ROIs were FOP1+43, OP1+OP2-3+PFcm, and OP4.

S1.10. Early auditory cortex

Cluster 10 contains five ROIs including the primary auditory cortex (A1), the lateral, medial, and parabelts (LBelt, MBelt, PBelt), and the retro-insular cortex (RI). These regions range in strength from 0.1390% to 0.4598%. Our algorithm suggests this cluster should contain two ROIs. PBelt (0.4598%) and RI (0.2081%) had no gradient in resting-state functional connectivity along the border, differing primarily in in myelination (however, PBelt does demonstrate stronger functional connectivity with a thalamic seed), so these two ROIs were merged (total strength 0.6679%). Similarly, A1 (0.1390%) and MBelt (0.1995%) demonstrated no gradient in resting-state functional connectivity (although these regions differed in myelination, activation in the motor-cue task, and function connectivity with a thalamic seed), so these ROIs were also merged. Finally, LBelt (0.2057%) is anatomical neighbours with both PBelt+RI and A1+MBelt and has a moderate FC gradient along borders with both. Therefore we merged LBelt with A1+MBelt (total strength 0.5442%) to achieve uniformity in strengths. Our final ROIs were therefore A1+MBelt+LBelt and PBelt+RI.

S1.11. Auditory association cortex

Cluster 11 contains eight ROIs including auditory complexes 4 and 5 (A4, A5), the dorsal/ventral anterior/posterior superior temporal sulcus (STSda, STSdp, STSva, STSvp), the superior temporal gyrus region a (STGa), and temporal region A2 (TA2). These regions range in strength from 0.2595% to 0.7172%. Our algorithm predicted the target number of ROIs in this cluster was five. This region was reasonably homogeneous in resting-state functional connectivity, with the primary FC gradient falling on the dorsal/ventral border of the superior temporal sulcus. The two weakest ROIs were STGa (0.2595%) and TA2 (0.3082%), which are anatomical neighbours which lie on the planum polare and superior temporal gyrus anterior to Heschl's gyrus, with main differences in terms of activation during tasks (Glasser et al., 2016). We therefore merged these ROIs (total strength 0.5768%). The next weakest logical pair of ROIs was the STSda (0.4461%) and STSdp (0.3976%), which are anatomical neighbours on the dorsal superior temporal sulcus and differed only in terms of myelination and activation during tasks, with no differences in resting-state functional connectivity. Therefore these ROIs were also merged (total strength 0.8437%). Finally, the border of the combined STSva+STSvp region differs markedly in resting-state functional connectivity compared to the remaining ROIs, and STSva has the lowest remaining strength (0.4396%), suggesting these ROIs should be merged (total strength 1.1429%). The resulting strengths of ROIs were in the range 0.5677% to 1.1429%. The resulting five ROIs were A4, A5, STSda+STSdp, STSva+STSvp, and STGa+TA2.

S1.12. Insular and frontal opercular cortex

In the original HCP-MMP atlas, the insular and frontal opercular cortex were very described in very high resolution, consisting of thirteen ROIs including area 52 (52), the parainsular cortex (PI), the insula granular (Ig), two posterior insular areas (PoI1 and PoI2), four frontal opercular areas (FOP2, FOP3, FOP4, and FOP5), the middle and anterior ventral insular areas (MI and AVI), the anterior agranular insular complex (AAIC), and the piriform cortex (Pir). However, due to the depth of this cluster, these ROIs had relatively low strength, ranging from 0.0724% to 0.2759%, with the exception of Pir which had a notably larger strength 0.6485%. Therefore our algorithm suggested this region should consist of only three ROIs.

Among the four frontal opercular areas, there is very little resting-state FC gradient, and differences are primarily in cortical thickness, gambling reward, math, and motor tasks (Glasser et al., 2016). Furthermore, in our analysis FOP2-3 were among the weakest ROIs (0.0925% and 0.0597% respectively), whilst FOP4-5 were among the strongest when Pir was excluded (0.2850% and 0.2427% respectively). We therefore merged these ROIs to form a single frontal opercular ROI (total strength 0.6799%).

Another group of ROIs that are largely homogeneous in terms of resting-state FC are Ig (strength 0.0678%), 52 (0.0679%), PoI1 (0.1193%), PoI2 (0.1273%), PI (0.1793%), and MI (0.1550%). This group of ROIs has a strong gradient in resting-state FC on the MI with AAIC and MI and the Ig border with FOP2, but internally displays relatively little FC gradient. The notable exception is a modest FC gradient between PoI2 and MI; however, in terms of both FC gradients and uniformity of ROI strengths, this merged ROI is the most appropriate placement for MI. It is additionally worth noting that 52 has a much stronger resting-state FC with a thalamic seed than the rest of this group. The total strength of this merged ROI was 0.7166%).

The final merged ROI therefore consisted of the remaining ROIs AAIC (0.0929%), AVI (0.1509%), and Pir (0.4528%), which make up the anterior region of the insular cortex (total strength 0.6966%). Unfortunately, in terms of resting-state FC, these regions are highly heterogeneous, both between and within the ROIs. However, due to the low resolution of MEG and the deep/weak nature of these regions, we propose that even a much higher resolution parcellation would be unlikely to identify these fine FC alterations as the insular cortex had some of the highest spatial extents of cross talk (Figure 4).

Our final three ROIs in cluster 12 were therefore FOP2+FOP3+FOP4+FOP5, Ig+52+PoI1+PoI2+PI+MI, and AAIC+AVI+Pir, with strengths ranging from 0.6799% to 0.7166%.

S1.13. Medial temporal cortex

In the original HCP-MMP atlas, the medial temporal region contained seven regions; however here we considered only six of these regions, excluding the hippocampal grey matter from the analysis. These six ROIs were three perihippocampal areas (PHA1, PHA2, PHA3), the peri-entorhinal/ectorhinal complex (PeEc), the entorhinal cortex (EC), and the presubiculum (PreS). The strengths of these ROIs were in the range 0.0911% to 0.5400%, and our algorithm suggested there should be two ROIs within this cluster. There was a large resting-state FC gradient throughout this region, so our motivation for merging ROIs was predominantly motivated by aiming towards uniform strengths. The first resulting ROI consisted of three perihippocampal areas (PHA1 0.1312%, PHA2 0.0911%, PHA3 0.1220%) and PreS (0.3791%; total strength 0.7234%). The second ROI consisted of the remaining regions EC (0.2818%) and PeEc (0.5400%; total strength 0.8218%). The resulting two ROIs in the cluster were Ec+PeEc, and PHA1+PHA2+PHA+PreS.

S1.14. Lateral temporal cortex

The lateral temporal cortex contains a total of nine ROIs, including two (TGd and TGv) in the temporal polar cortex, three (TF, TE2a, and TE2p) in the inferior temporal sulcus and gyrus, and four (TE1a, TE1m, TE1p, and PHT) in the middle temporal gyrus. The algorithm suggested nine ROIs was optimum, and hence cluster 14 was unaltered in the reduced atlas.

S1.15. Temporo-parieto-occipital junction

Cluster 15 contains five ROIs, including three on the temporo-parieto-occipital junction (TPOJ1, TPOJ2, and TPOJ3), the Perisylvian language area (PSL), and the superior temporal visual area (STV). The optimum number of ROIs was four. This cluster forms a strip of cortex with ROIs arranged linearly, and hence there are a limited number of possible combinations to merge. There is strong functional connectivity between areas TPOJ1-3 (0.7372%, 0.8010%, 0.3686% respectively) and STV (0.6611%) Glasser et al. (2016), so it is natural to merge two of these regions. Since TPOJ3 had notably smaller strength than the other regions in this cluster, we merge this ROI with its neighbour TPOJ2 (total strength 1.1696%). The resulting range in strengths was therefore 0.6532% (PSL) to 1.1696%. The resulting four ROIs were TPOJ1, TPOJ2+TPOJ3, PSL, STV.

S1.16. Superior parietal cortex

The superior parietal cortex contains ten ROIs, including medial and lateral area 7P (7Pm, 7PL), medial and lateral area 7A (7Am, 7AL), area 7PC (7PC), and five intraparietal/lateral intraparietal areas (LIPv, LIPd, VIP, MIP, AIP). These ROIs ranged in strengths from 0.2793% to 0.9229%. Our algorithm suggested the optimum number of ROIs was eight, so based primarily on strengths and FC gradients reported by Glasser et al. (2016), we chose to merge the lateral and medial areas 7P (7PL 0.4132%, 7Pm 0.3583%; total 0.7715%) and the dorsal/ventral LIP (LIPd 0.2608%, LIPv 0.5107%; total 0.7715%). The resulting range of ROI strengths was 0.5440%-0.8239%. The resulting eight ROIs were 7Pm+7PL, 7Am, 7AL, 7PC, LIPv+LIPd, VIP, MIP, AIP.

S1.17. Inferior parietal cortex

The inferior parietal cortex contains 10 ROIs, including a transitional area PGp, three areas on the intraparietal sulcus (IP0, IP1, and IP2), three areas corresponding to a subdivision of the classical PF (PF, PFt, and PFop), and three nodes of the task positive network (PFm, PGi, PGs). The initial iterate of the algorithm predicted cluster 17 should contain 13 ROIs, so like clusters 1, 2, and 6, this cluster is actually over represented in the reduced parcellation. Accounting for the fact we do not split ROIs, the final iterate of the algorithm suggested ten ROIs was optimum, and hence cluster 17 was unaltered in the reduced atlas.

S1.18. Posterior cingulate cortex

The posterior cingulate cortex contains 14 ROIs, which Glasser et al. (2016) described as functionally distinct, and grouped based upon geographical proximity rather than functional similarity. There are large resting-state functional connectivity gradients on the borders of many ROIs within this cluster, so uniform strengths were prioritised in merging ROIs for cluster 18. Strengths of the ROIs ranged from 0.0930% to 0.5219%, excluding the parietal occipital sulcus area 2 (POS2) which had a strength of 0.9609%. Our algorithm suggested the optimum number of ROIs was five.

The first merged ROI consisted of transitional areas between the early visual cortex and posterior cingulate association cortex found on the anterior bank of the parietal-occipital sulcus. These ROIs were the dorsal visual transitional (DVT 0.5219%) and prostriate (ProS 0.1046%) cortices, and first parietal-occipital sulcus (POS1 0.2919; total strength 0.9184%). DVT and ProS differ primarily in myelination and activation during a number of task Glasser et al. (2016). Although there exists a modest difference in FC between these regions and POS1, based on uniformity of ROI strengths this was the most appropriate region with which to merge POS1.

Furthermore, Glasser et al. (2016) separated area 31 into three ROIs, namely anterior 31 (31a; 0.1366%) and dorsal/ventral posterior 31 (31pd/31pv; 0.1381%/0.1027% respectively). Borders between these regions were primarily due to activation in tasks, although modest gradients in myelination and resting-state functional connectivity were also reported. Therefore, for our second ROI we merged these three ROIs into a single area 31. Area 7m (0.3877%), which shares an anterior border with 31pd and differs primarily in tasks (although a small difference in FC between these regions was reported), was also merged with these areas (total strength 0.7651%).

Similarly to area 31, Glasser et al. (2016) subdivided area 23 into the dorsal and ventral 23ab (d23ab/v23ab; 0.0930%/0.0997%), 23c (0.2823%), and 23d (0.1375%). With the exception of 23c, these areas differed only modestly in resting-state functional connectivity. However, there was a strong gradient in resting-state functional connectivity along the border of 23c the rest of 23. We therefore merged d23ab, v23ab, and 23d, but did not include 23c in this ROI. Instead, 23c and the precuneus visual area (PCV; 0.4188%) were merged (total strength 0.7011%), based on the fact these ROIs did not differ in resting-state functional connectivity, but did have a strong gradient along their borders with the rest of cluster 18.

Two ROIs remain which have not been discussed in detail, namely parieto-occipital sulcus area 2 and the restrosplenial complex (RSC; 0.3021%). Since POS2 had a notably larger strength than any other ROI, we did not merge this with any other ROIs. RSC is a narrow strip along the inferior edge of cluster 18, and differs strongly in functional connectivity with all anatomical neighbours in the cluster. However, due to the low strength of this ROI, it is required to merge with another region in order to maintain uniformity.

Based on geographical location and uniformity of ROI strengths, we merge RSC with our merged area 23 (total strength 0.6323%).

The resulting range of strengths for cluster 18 were therefore 0.6323% to 0.9609%. The five resulting ROIs for this cluster were DVT+ProS+POS1, d23ab+v23ab+23d+RSC, 31a+31pd+31pv+7m, PCV+23c, and POS2.

S1.19. Anterior cingulate and medial prefrontal cortex

Cluster 19 had 15 ROIs in the original HCP-MMP parcellation. Strengths of ROIs within this cluster ranged from 0.0979% to 0.8146%. Our algorithm suggested this cluster should contain six ROIs.

This cluster can largely be split into two sub-clusters based on a strong resting-state FC gradient (Glasser et al., 2016). Posterior to this border largely corresponds to the posterior part of the anterior cingulate cortex split into three bands, namely area 33pr (0.2594%), area 24pr and posterior area 24 (a24pr 0.1057%; p24pr 0.1437%; p24 0.2661%), and area 32pr (a32pr 0.1445%; p32pr 0.1819%). Within this sub-cluster of regions, there was very little gradient in FC. We therefore merge these regions into two ROIs based primarily on cingulate band, resulting in the ROIs 33pr+a24pr+p24pr (total strength 0.5088%) and p32pr+a32pr+p24 (total strength 0.5925%). The only deviation from the rule of merging by band is the inclusion of p24 in the latter ROI as opposed to the former. This was motivated by homogeneity of ROI strengths, since inclusion of p24 in the former ROI would result in highly heterogeneous strengths of 0.7749% and 0.3264% respectively. The modest gradient in FC between p24 and a24pr further justifies splitting the band corresponding to area 24 and 24pr (Glasser et al., 2016).

Anterior to the FC border splitting cluster 19 is the medial prefrontal cortex and the anterior parts of the cingulate bands corresponding to areas 24 and 32. A second, weaker FC gradient and anatomical location justifies further splitting this sub-cluster into superior and inferior sections. The superior ROIs include the superior medial prefrontal areas 8BM (0.5952%) and 9m (0.8146%) and the cingulate dorsal area 32 (d32, 0.2387%). The small FC gradient between 8BM and d32, and the larger FC gradient between these regions and area 9M - combined with the desire for homogeneous ROI strengths - justify the merging of 8BM+d32 (total strength 0.8339%), and leaving 9m unmerged.

The inferior part of this sub-cluster consists of four cingulate ROIs p32 (0.1606%), s32 (0.0979%), a24 (0.2893%), and 25 (0.2614%), and two subdivisions of the medial surface of the orbital prefrontal cortex, 10r (0.2419%) and 10v (0.4921%). There are only small differences in resting-state FC between these ROIs, so the ROIs are merged according to anterior cingulate/inferior medial prefrontal boundaries; i.e. the resulting two ROIs are p32+s32+a24+25 (total strength 0.8092%) and 10r+10v (total strength 0.7340%). It should however be noted that there was a strong gradient in FC around all borders of p32 (Glasser et al., 2016), making the choice of merge reasonably arbitrary.

The resulting eight ROIs in cluster 19 therefore ranged in strength from 0.6957% to 0.8339%, and were a24pr+p24pr+33pr, a32pr+p32pr+p24, 9m, 8BM+d32, p32+s32+a24+25, and 10r+10v.

S1.20. Orbital and polar frontal cortex

The orbital and prefrontal cortex consisted of 11 ROIs, including the orbitofrontal and posterior orbitofrontal complexes (OFC, pOFC), areas 13l and 11l, three subdivisions of area 47 (47s, 47m, a47r), and four subdivisions of area 10 (10pp, a10p, p10p, and 10d). These ROIs ranged in strength from 0.1377% to 0.8773%. Our algorithm suggested seven ROIs for this region. Two of the weakest ROIs in this cluster were subdivisions of area 47, 47m (0.1377%) and 47s (0.2787%). Glasser et al. (2016) reported a small difference in functional connectivity between these regions, but the primary difference was in myelination. We therefore merged these regions (total strength 0.4164%). Interestingly, the remaining subdivision of 47, a47r (0.8773%) was the strongest ROI, so this was not merged with 47m+47s.

13l (0.2558%) was the next weakest ROI. We merged 13l with 11l (0.5481%; total strength 0.8039%), forming a complex which lies lateral to the orbitofrontal complexes (Glasser et al., 2016). We additionally merged the four subdivisions of area 10 into two pairs of subdivisions; the anterior/posterior area 10p (a10p/p10p; 0.4310%/0.5465%) formed one subdivision (total strength 0.9775%), whilst 10d (0.5849%) and 10pp (0.3628%) formed the other subdivision (total strength 0.9477%). The range of strengths in the

resulting seven ROIs were 0.3554% to 0.9775%, which were OFC, pOFC, 13l+11l, 47s+47m, a47r, 10pp+10d, and a10p+p10p.

S1.21. Inferior frontal cortex

The inferior frontal cortex consists of eight ROIs including areas 44, 45, two subdivisions of 47 (47l, p47r), and four subdivisions of the inferior frontal sulcus (IFJp, IFJa, IFSp, and IFSa). Excluding the weak anterior/posterior IFJ regions (IFJa/IFJp; 0.2552%/0.1419%), these ROIs were largely heterogeneous in strength, ranging from 0.4739% to 0.6985%. Our algorithm suggested that this cluster should contain five ROIs, so IFJa and IFJp were merged with the neigbouring IFSp (0.4739%; total strength 0.8710%). Furhtermore the two subdivisions of area 47 were merged (total strength 1.0518%). The resulting range of strengths were 0.6404% to 1.0518%. The five ROIs in cluster 21 were therefore 44, 45, 47l+p47r, IFJa+IFJp+IFSp, IFSa.

S1.22. Dorsolateral prefrontal cortex

The dorsolateral prefrontal cortex contains 13 ROIs, including the superiod frontal language are (SFL), two transitional areas between areas 6 and 8 (s6-8 and i6-8), a subdivision of area 8 into four areas (8BL, 8Ad, 8Av, and 8C), a subdivision of area 9 into two areas (9p and 9a), and four areas in the central portion of the dorsolateral prefrontal region (9-46d, 46, a9-46v, and p9-46v). The algorithm suggested 13 ROIs was optimum, and hence cluster 22 was unaltered in the reduced atlas.

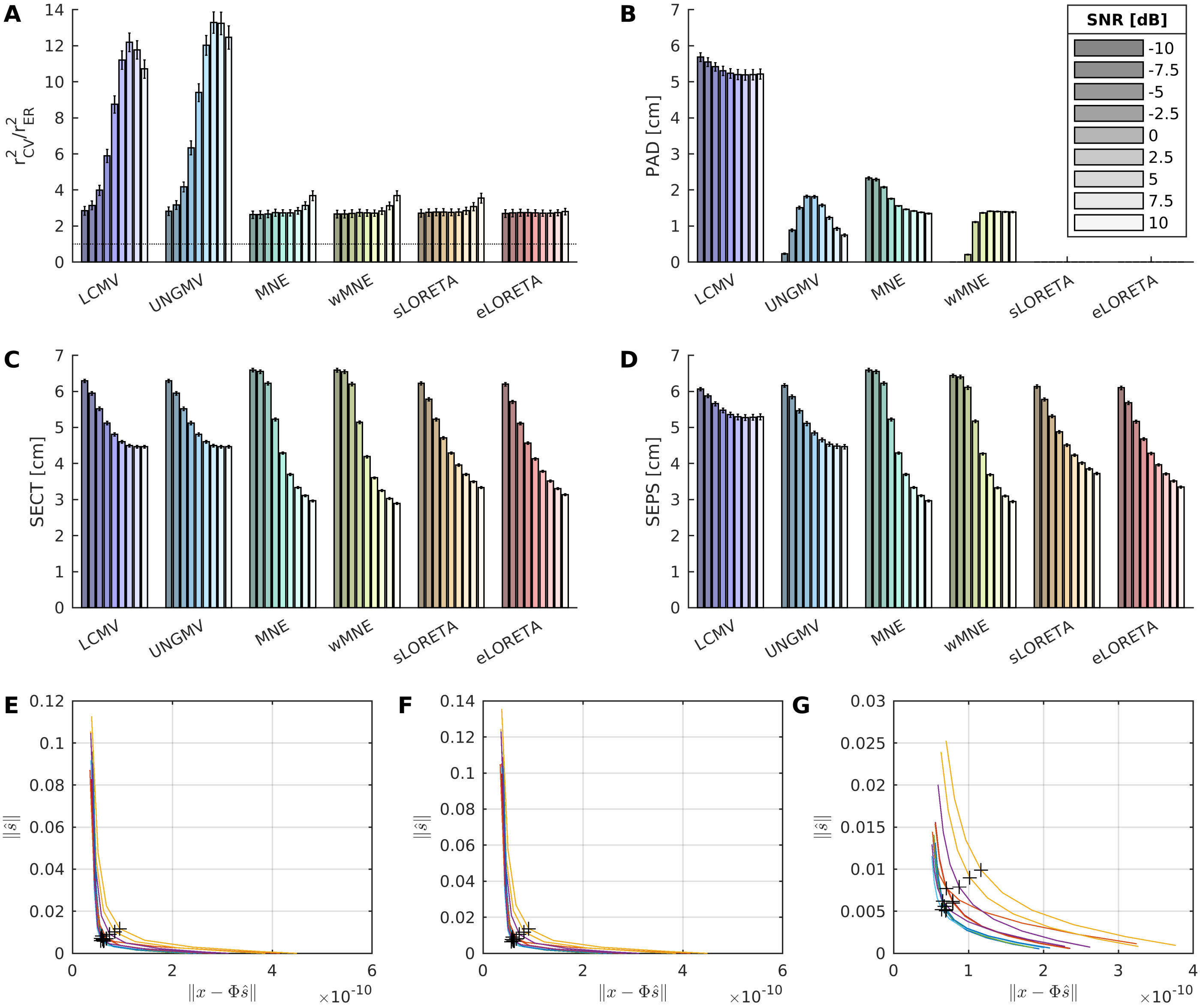

Supplementary Figure S1: **Choosing regularization parameter based on predicted SNR.** (A-D) r^2_{CV}/r^2_{ER}, PAD, SECT, and SEPS respectively, showing how these values vary as the predicted SNR (and therefore regularization parameter) is varied. In A, the dotted black line shows $r^2_{CV}/r^2_{ER} = 1$; all methods fall above this line for all SNRs, meaning the resting-state data explains more variance to empty room. This suggests none of the methods completely overfit to the data. (E-G) L-curves for the least squares minimum norm type estimates MNE, wMNE, and eLORETA respectively. Colours represent participants. The L-curves suggest that SNR of approximately 2.5 dB (black crosses show locations corresponding to 2.5 dB) is optimum. This is also an acceptable value for the beamformers, since it is approximately the location of peak r^2_{CV}/r^2_{ER} and PAD (for UNGMV). This justifies our use of SNR=2.5 dB throughout this study.

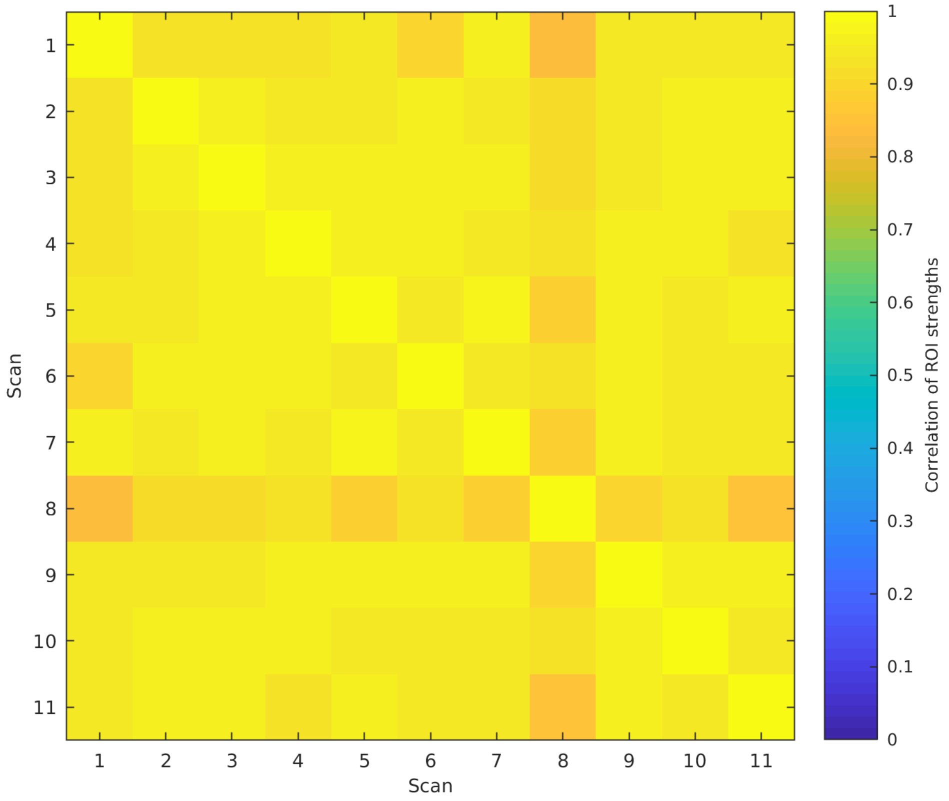

Supplementary Figure S2: **ROI strengths are highly consistent across subjects**. The reduction of the HCP-MMP parcellation, described in section 2.3 and shown in Figure 6, made use of the average ROI strength for each ROI over 11 participants. To ensure these values were consistent across subjects, i.e. the same areas had large/small strength in all subjects, the distributions of ROIs strengths were correlated. All pairs of subjects had a correlation > 0.83, suggesting the spatial patterns of ROI strengths are highly consistent across subjects.

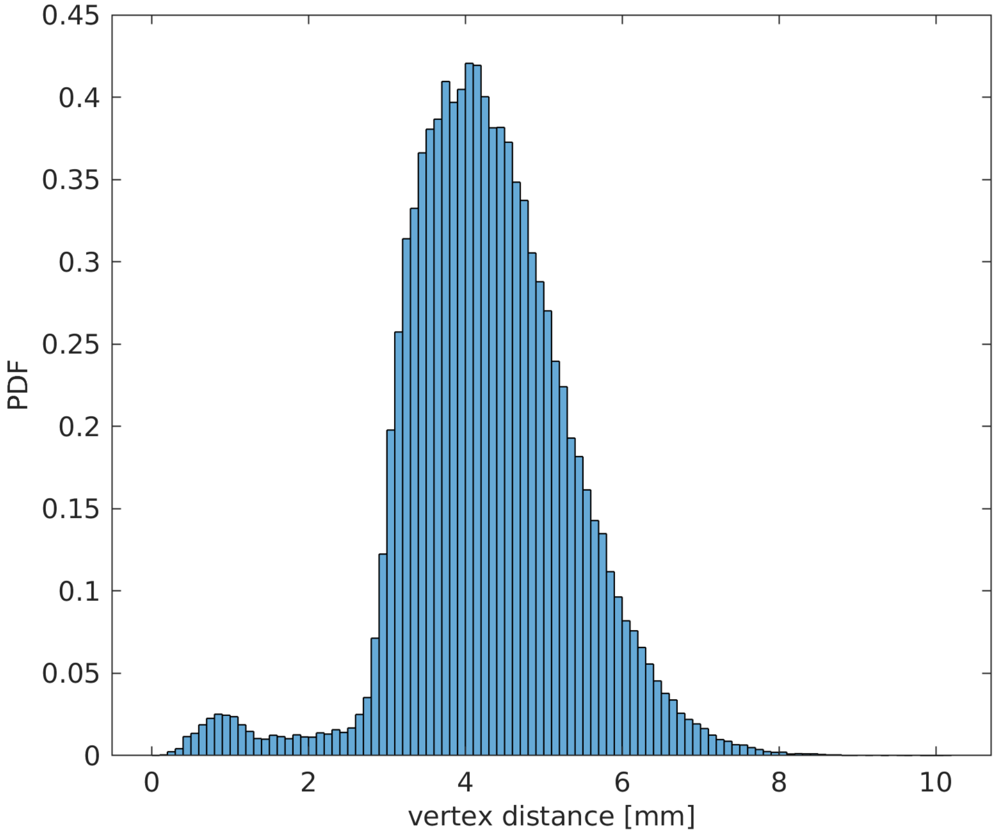

Supplementary Figure S3: **Distribution of distances between neighbouring vertices in cortical mesh.** For each participant, 10,000 pairs of neighbouring vertices in the cortical mesh were randomly sampled without repetition and the distance between pairs calculated. Shown here is a histogram, normalized to a probability distribution function (PDF), of distances over the resulting 110,000 distances sampled.

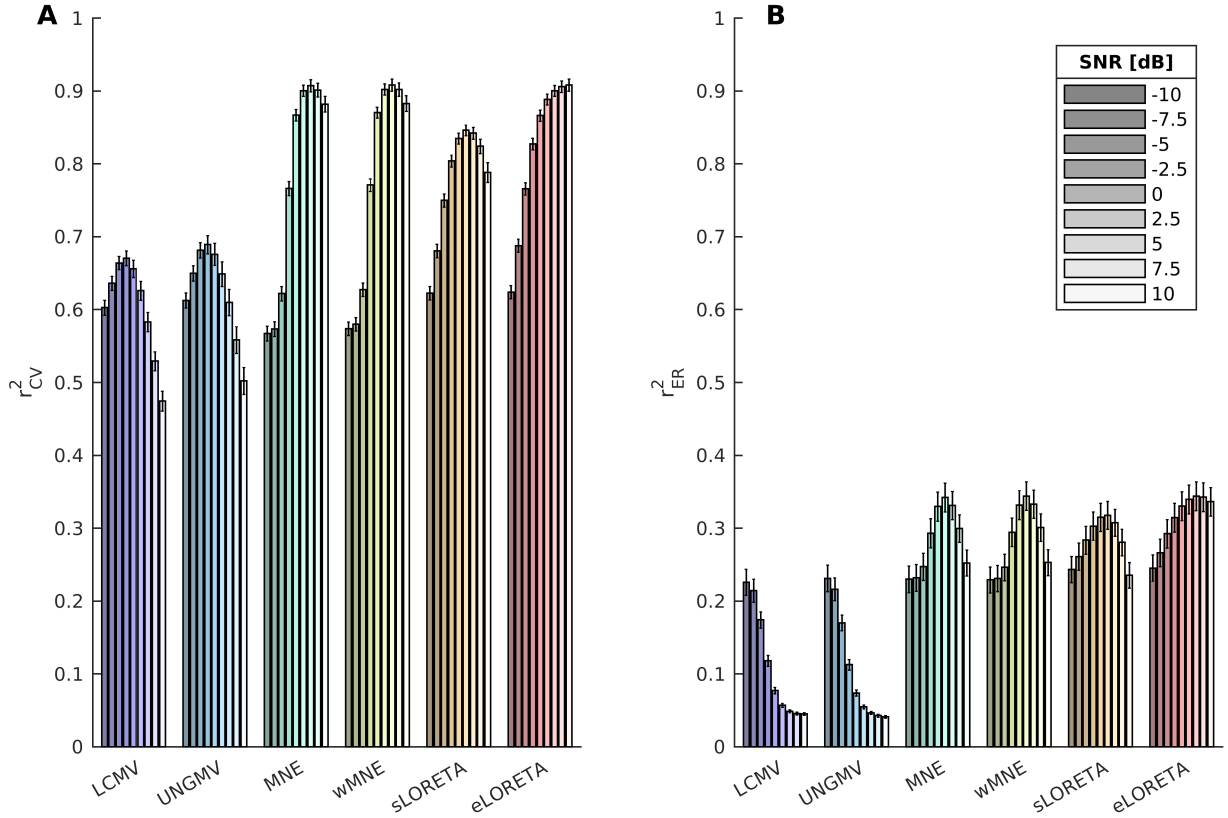

Supplementary Figure S4: **Raw values of r^2_{CV} and r^2_{ER} as predicted SNR is varied.** Throughout the study, values of the ratio r^2_{CV}/r^2_{ER} are shown. Here we show the raw values of each of these metrics, as opposed to the ratio.

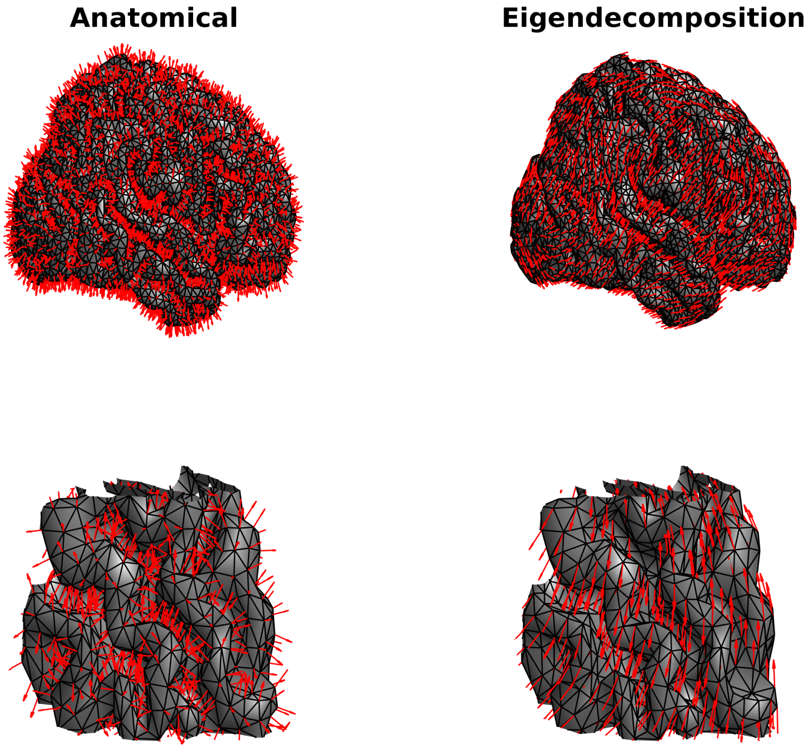

Supplementary Figure S5: **Example of dipole orientations for a single participant using different orientation constraints.**

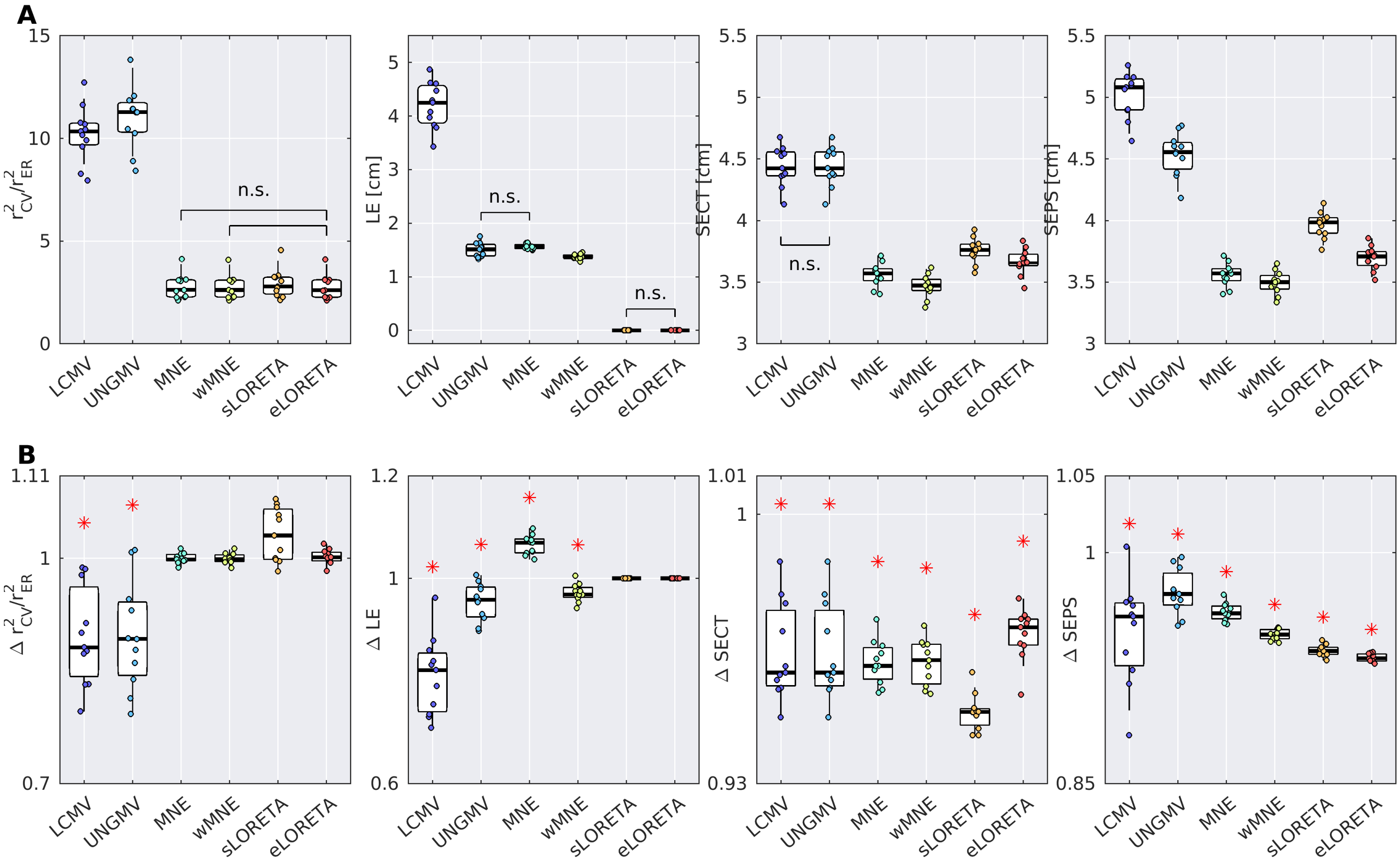

Supplementary Figure S6: **Comparison between orientation constraints.** (A) r^2_{CV}/r^2_{ER}, PAD, SECT, and SEPS for each algorithm in the eigendecomposition case. For all figures, group-wise analyses demonstrated a significant effect of algorithms. Pairwise analyses identified significant differences for all pairs except those marked as non-significant (n.s.), following false discovery rate correction. (B) Each of the measures in A, shown as a ratio of eigendecomposition to anatomical orientation constraints. Algorithms for which there was a significant pairwise difference between orientation constraints following false discovery rate correction are marked by an asterisk. Values for whiskers, boxes, etc. are as described in Figure 3.

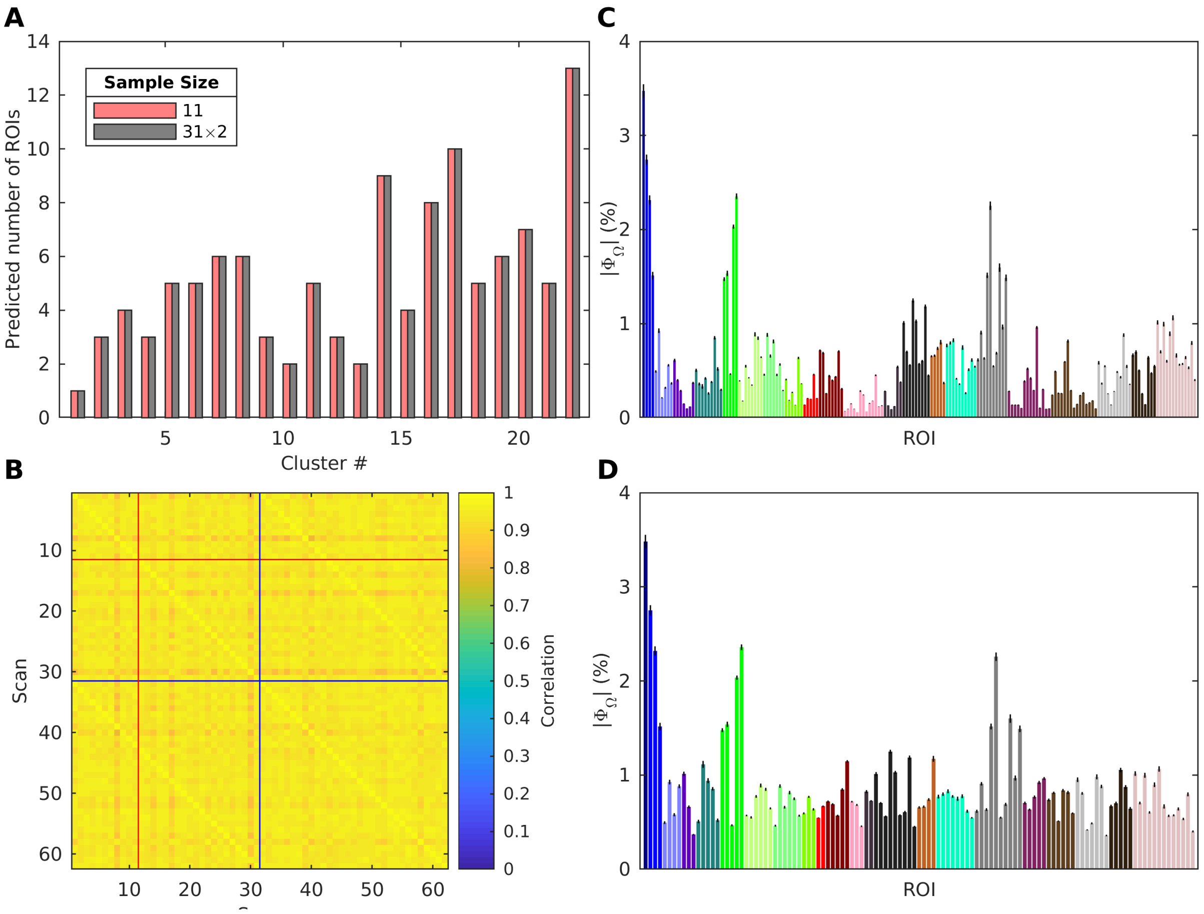

Supplementary Figure S7: **Validation of the HCP-230 atlas in a larger cohort.** The results presented in Figure 6 show the reduction of the HCP atlas to 230 ROIs based on the same 11 participants used to compare algorithms throughout the study. Here, the atlas is validated on a larger cohort consisting of an additional 20 participants (31 total), using Polhemus digitisers to generate head positions from two different resting state sessions for each participant (resulting in a total of 31x2=62 leadfield matrices). (A) The predicted number of ROIs in each macro-scale cluster of regions are equal in the original 11 participants (red) and the larger cohort (grey). (B) Correlation between strengths of ROIs across participants and sessions. The red line shows the 11 original participants, therefore the red square in the top left is equivalent to Figure S2. The blue lines separate sessions, i.e. scans 1-31 are from the first session, scans 32-62 are a 2nd session from the same participants. (C-D) Reproduction of Figure 6A and D respectively for the larger cohort. C shows ROI strengths for the original atlas, D shows the same for the downsampled atlas.
